# Two-Dimensional Nanostructures for Electrochemical Biosensor

**DOI:** 10.3390/s21103369

**Published:** 2021-05-12

**Authors:** Reem Khan, Antonio Radoi, Sidra Rashid, Akhtar Hayat, Alina Vasilescu, Silvana Andreescu

**Affiliations:** 1 Department of Chemistry and Biomolecular Science, Clarkson University, Potsdam, NY 13699, USA; rekhan@clarkson.edu; 2 National Institute for Research and Development in Microtechnology—IMT Bucharest, 126A Erou Iancu Nicolae Street, 077190 Voluntari, Romania; antonio.radoi@gmail.com; 3 IRCBM, COMSATS University Islamabad, Lahore Campus, Lahore 54000, Pakistan; srasheed075@gmail.com (S.R.); akhtarhayat@cuilahore.edu.pk (A.H.); 4 International Centre of Biodynamics, 1B Intrarea Portocalelor, 060101 Bucharest, Romania; avasilescu@biodyn.ro

**Keywords:** 2D nanostructures, hierarchical structure, MXenes, graphene, biosensors, field analysis, TMDs

## Abstract

Current advancements in the development of functional nanomaterials and precisely designed nanostructures have created new opportunities for the fabrication of practical biosensors for field analysis. Two-dimensional (2D) and three-dimensional (3D) nanomaterials provide unique hierarchical structures, high surface area, and layered configurations with multiple length scales and porosity, and the possibility to create functionalities for targeted recognition at their surface. Such hierarchical structures offer prospects to tune the characteristics of materials—e.g., the electronic properties, performance, and mechanical flexibility—and they provide additional functions such as structural color, organized morphological features, and the ability to recognize and respond to external stimuli. Combining these unique features of the different types of nanostructures and using them as support for bimolecular assemblies can provide biosensing platforms with targeted recognition and transduction properties, and increased robustness, sensitivity, and selectivity for detection of a variety of analytes that can positively impact many fields. Herein, we first provide an overview of the recently developed 2D nanostructures focusing on the characteristics that are most relevant for the design of practical biosensors. Then, we discuss the integration of these materials with bio-elements such as bacteriophages, antibodies, nucleic acids, enzymes, and proteins, and we provide examples of applications in the environmental, food, and clinical fields. We conclude with a discussion of the manufacturing challenges of these devices and opportunities for the future development and exploration of these nanomaterials to design field-deployable biosensors.

## 1. Introduction

The development of practical biosensors that can detect low concentrations of analytes inexpensively and rapidly has been the subject of intensive investigations with the goal to advance measurement sciences in fields such as clinical diagnostic, environmental monitoring, and food safety [1,2]. The success of biosensors as measurement tools relies on achieving the robustness and accuracy necessary to compete with conventional analytical tools for field analysis. The key requirement is to design an optimum sensing surface that can stabilize biological recognition molecules and be interfaced with physical transducers that convert the biorecognition into a quantifiable signal [3]. Therefore, the selection of an appropriate sensing material possessing both recognition and transduction functions is essential in achieving the needed performance. Varieties of materials have been used to create this interface including different forms of carbon, metals, and metal oxides [4]. Nanostructured materials introduced more than a decade ago have been combined with biological reagents to integrate the optical, electronic, and catalytic properties of nanomaterials with the biorecognition capabilities of the bio-entities, resulting in improved performance [5,6]. In most conventional designs, nanostructured materials are deposited in monolayered configurations, which limit the surface to the immobilization of a few biomolecules. The recent development of layered and precisely designed hierarchical nanostructures provides opportunities for creating multiscale structures with controlled functions and improved electrical, optical, and mechanical properties.

Research into elemental layered structures can be traced back to the 1930 with the pioneering work of Langmuir, who laid the foundations of surface science. Since then, surface scientists have studied the formation and properties of a large number of layered entities, some of which have shown potential in the sensing field [7]. To be used in sensing, these materials should possess high electronic, catalytic, and mechanical properties; they should be biocompatible and amenable to functionalization with biological molecules. In most cases, the as-synthesized structures need additional activation to generate the functional groups needed to attach biomolecules for achieving selective recognition and sensing. In some cases, the layered materials have the desired mechanical stability but lack sufficient electronic, optical, or surface properties necessary for field functional devices. Therefore, they have been interfaced with materials possessing these functions, creating a variety of more complex hybrid structures. The physicochemical properties and applications of hierarchical nanostructures in various fields have been reported in several recent reviews [8,9,10]; here, we focus primarily on their uses in the biosensing field.

Layered or multidimensional nanomaterials have gained significant attention in the biosensing field due to their high surface area, functionalized surface, and quantum size effect [11]. Their implementation in the biosensing field requires modification in order to impart selectivity and sensitivity for the detection of analytes within useful limits [12]. Other requirements are related to the challenge of scalable production of the structures themselves and the integration of sensing functions into the actual device [13]. The multifunctionality of 2D nanomaterials provides characteristics such as tunable structure and a large number of active sites along with an ultra-thin planar structure [14] that can be beneficial for detection and transduction. This ultra-thin assembly makes 2D nanomaterials more sensitive to external perturbations [15], and therefore, higher detection sensitivities can be achieved. Two-dimensional (2D) layered and multidimensional nanomaterials are commonly prepared by Top–Down Liquid Phase Exfoliation (TDLPE) or by Bottom–Up Surfactant Direct Growth (BUSD) methods where they can easily restack and lead to the formation of dense platforms [16]. To prevent uncontrollable restacking or aggregation, which can affect the biosensor’s performance, hierarchical structural architectures [17] can be designed by incorporating other dimensional entities [18] in between the layers. The resulting hybrid structures show not only the inherent characteristics of the parent 2D material but also functions such as porous structures with bulk void spaces for bioimmobilization or added catalytic sites for enhanced transduction [16]. A good hierarchical organization is expected to be mechanically adjustable and stable, permeable, having good pore volume, low structural density, and allow fast mass transport [19]. Thus, the ability to control these parameters is essential when using these materials for the design of field applicable biosensors.

**Figure 1:**
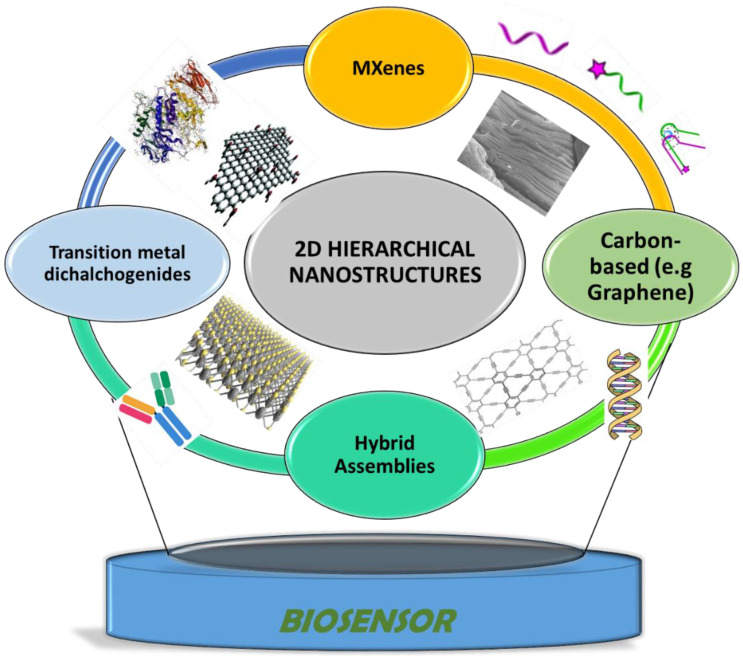
Hierarchical nanostructures used as electrode materials and bioimmobilization support for the construction of electrochemical biosensors discussed in this paper.

In this review, we provide an overview of the different types of 2D nanomaterial-based hierarchical assemblies (carbon-based, transition metal dichalcogenides, MXenes, and hybrid nanostructures. Figure 1 shows the potential for the design of electrochemical biosensors for field analysis. Different fabrication strategies involving various 2D nanomaterials are discussed along with several examples of nanostructured architectures that have been used a building blocks to develop biosensors for the detection of analytes of interest in the clinical, environmental, and food analysis fields. Finally, challenges and opportunities for future developments to solve practical challenges in emerging areas, including the realization of flexible and wearable devices based on multidimensional materials, are presented.

## 2. 2D Nanostructures: Synthesis, Properties, and Integration in Biosensing Design

Hierarchical nanostructures developed from 1D or 2D building blocks have attracted considerable interest due to their physical, optical, and electronic properties [7,8,9,10]. Of these, improvement in electron transfer kinetics for electrochemical devices and their use as support for electroactive species and for the immobilization of biological molecules are the most relevant [20]. This class of materials is large and includes graphene (G) and graphene-like materials, e.g., graphidyne (GDY) [21] transition metal oxides, transition metal dichalcogenides (TMDs), and MXenes [22]. Their properties are attributed to a large density of surface-active sites providing optimum configurations for biological sensing. These properties are summarized in the following sections.

### 2.1. 2D Carbon-Based Nanostructures: Graphene, Graphynes, and Graphidyne

Carbon is one of the most used materials for electrochemical sensors and biosensors due to its availability, chemical inertness, wide potential window, and lower noise compared to other types of electrode materials, e.g., metal electrodes [23]. The ease of fabrication in different sizes and configurations and the reduced cost make carbon-based low-dimensional nanostructures particularly appealing. Additional features such as high mobility of the charge carriers, electrical conductivity, and large surface area make them excellent choices for electrochemical sensing. Examples of 2D carbon (C) nanomaterials are graphene, graphynes, and graphidyne, among which graphene is the most well-known and most studied 2D material. Graphene has a hexagonal sheet-like structure in which all the C atoms are sp^2^ hybridized. Graphyne has a structure similar to graphene but contains a mixture of sp- and sp^2^-hybridized C atoms that form an interconnected network of benzene rings and acetylene bonds. Due to the presence of this mixed hybridization, the graphyne structure is slightly distorted from a hexagonal array to a triangular geometry. However, it is not a real triangular structure but a strained hexagon, which looks like a triangle [21]. Graphidyne (GDY), first proposed in 1987, shares several similarities with graphene including the typical 2D structure, but unlike graphene, GDY is a network of interconnected benzene rings each joined together by diacetylenic linkages where two C-C triple bonds are connected by a single C-C bond.

The discovery of graphene-based materials and their properties paved the way for developing new 2D layered and non-layered materials. Graphene-based materials have good electrical conductivity, large theoretical specific surface area up to 2630 m^2^g^−1^, high thermal conductivity, high young’s modulus, and optical transmittance. Graphene oxide has emerged as a precursor of graphene-based materials. Conventionally, the graphene is exfoliated from graphene oxide with the aid of stirring or mild sonication. Since graphene oxide is a good insulator due to the functional groups’ presence, deoxygenation is performed to recover its conducting properties, and the resulting product, reduced graphene oxide (rGO), is the typical material used for biosensing design [24].

**Figure 2:**
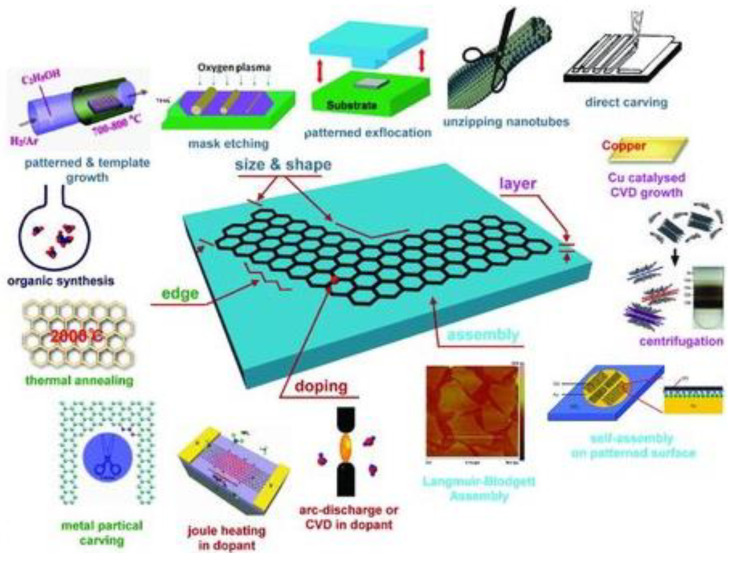
Various current techniques to synthesis graphene with controlled sizes and shapes, edges, and layers [30].

Figure 2 summarizes the various top–down and bottom–up approaches that have been explored to synthesize graphene. The exfoliation of graphene can be performed by mechanical, thermal reduction, chemical vapor deposition, physical vapor deposition, and plasma etching. The simplest and easiest method is to use mechanical exfoliation, or the “scotch-tape” method, which was first reported when isolating graphene [25]. The method allows obtaining atomically thin crystal sheets from layered materials, producing 2D nanomaterials having high crystal quality and macroscopic continuity [26]. Mechanical exfoliation is the fastest method to obtain 2D materials, but the technique is not scalable for large-scale production. Exfoliation into colloidal solutions assisted by sonication and using chemical intercalants is another method for the large-scale production of single and layered 2D materials. For example, high-yield dispersions (up to 0.01 mg mL^−1^) of pristine nano-flakes of graphene were obtained using *N*-methylpyrrolidone (NMP) as a liquid exfoliating environment [27]. Solvents with a surface tension (γ) of ≈40 mN/m, such as NMP, (γ = 40 mN/m), *N*,*N*’-dimethylformamide (DMF, γ = 37.1 mN/m), γ-butyrolactone (GBL, γ = 35.4 mN/m), and *ortho*-dichlorobenzene (o-DCB, γ = 37 mN/m), have been used for the exfoliation of graphite into graphene [28]. Recently, natural amino acids (e.g., alanine, glycerine, etc.) were proposed as intercalants for graphene exfoliation, due to their ability to deliver stable aqueous dispersions (32 mg mL^−1^) of typical few-layered nanostructures, corresponding to 2–5 layers of graphene [29].

The properties of graphene that makes this material of interest for biosensing include (i) the ability to interact with biomolecules via π–π interactions, (ii) to be chemically functionalized to immobilize specific molecular receptors onto their surface [31], and (iii) providing a suitable interface with various transduction modes. Particularly in electrochemical biosensors, the conjugate structure of graphene facilitates the charge transfer between the biomolecule and the transducer, thus increasing the sensitivity of the biosensor. However, several features can directly affect the sensing performance. For example, the synthesis route of graphene or its derivatives (GO, rGO), batch to batch variation, the extent of surface functional groups in GO and rGO, the orientation between graphene sheets and the bioreceptor, the number of layers, and the oxidation state of graphene, GO, and rGO can cause differences in the sensitivity and selectivity of the developed sensor [32].

Hao et al. reported an enzymatic glucose biosensor based on a graphene laminated electrode [33]. Although the surface functional groups and defects in graphene oxide are beneficial for biomolecule immobilization, the electrical conductivity and charge transfer properties of graphene can be compromised upon oxidation. To alleviate this problem, graphene oxide was used along with the edge-functionalized graphene (FG) to maximize the electrochemical performance of the sensor. The GO/FG structure provided enhanced charge transfer, higher detection sensitivity 46.71 μA∙mM^−1^∙cm^−2^, and high enzyme (glucose oxidase GOx) loading i.e., 3.80 × 10^−9^ mol cm^−2^. The increased performance of the GO/FG-based glucose sensor was attributed to the oxygen-rich surface of GO, high electron transfer of FG, and the availability of higher surface area for maximum loading of GOx [33]. GOx is considered to be a standard enzyme for developing glucose biosensors. It has relatively high selectivity for glucose as compared to that of other glucose enzymes such as glucose−1-dehydrogenase and hexokinase. Additionally, GOx is inexpensive, readily available, and can withstand greater pH variations, which ultimately leads to applications under less stringent conditions [34]. Furthermore, Wu et al. reported a GDY/tyrosinase-based biosensing system for the detection of bisphenol A (BPA). For the sensor fabrication, GCE was modified with GDY, and in the next step, a previously mixed dispersion of tyrosinase and chitosan was drop casted on the modified transducer. The response of the GDY biosensor toward the BPA was linear over a working range of about one order of magnitude (1.0 × 10^−7^ M–3.5 × 10^−6^ M) with a high sensitivity of 2990.8 mA cm^−2^ M^−1^ and a detection limit of 24 nM. This biosensor performed better for BPA detection, as compared to CNTs and graphene-based biosensors reported in the literature [35].

### 2.2. MXenes

**Figure 3:**
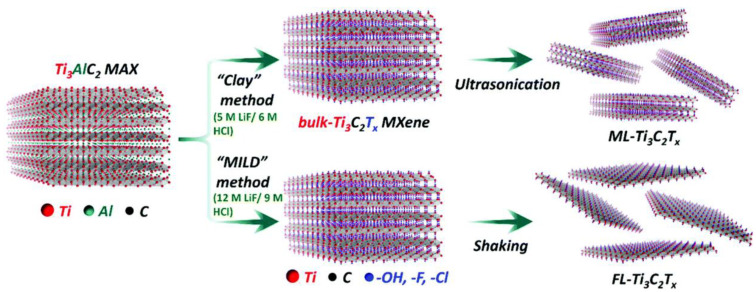
Scheme representing the synthesis route to prepare bulk, ML- and FL-Ti_3_C_2_Tx from the parent Ti_3_AlC_2_ MAX phase [43].

Among the family of 2D nanomaterials, MXenes are the latest and largest reported class of materials possessing high metallic conductivity, hydrophilicity, and high biocompatibility, which makes them interesting candidates for the design of electrochemical biosensors [22]. MXenes (Figure 3) are formed by the selective etching of ‘A’ layers from their corresponding MAX phases (i.e., M_n+1_AX_n=1;2;3._, where M represents an early transition metal (Sc, Ti, Zr, V, Cr, Mn, Nb, Hf, Ta, Mo), A is usually an element from group 12 to 16 of the periodic table (Cd, Al, Si, P, S, Ga, Ge, As, In, Sn, Tl, Pb), and X is either carbon (C), nitrogen (*N*), or both) [36]. MAX phases are different from graphite and other layered materials where the layers are held together by weak van der Waals forces, while a typical MAX phase is composed of a strong M-X bond that possesses a mixed metallic–covalent character and a relatively weaker M-A bond. Due to this mixed chemical bonding, different synthetic approaches are being used for the chemical exfoliation of MAX phases, which results in the formation of the corresponding MXene.

The most common approach is wet chemical etching using hydrofluoric acid (HF). In a typical procedure, the MAX phase is immersed in HF for a certain period of time. Due to the difference in the chemical bonding of M-A and M-X elements, these layers reacted differently toward HF, hence resulted in the selective etching of the A layer out of the MAX phase [37,38]. Initially, this procedure was applied for the synthesis of Ti_3_C_2_ from the parent MAX Ti_3_AlC_2_ [39], but later, various other types of MXenes have also been synthesized using HF [36,39,40]. The reaction parameters such as the concentration of HF and reaction time depend on the type of MAX phase used. The wet chemical etching procedure leads to the functionalization of MXenes surface with -O, -OH, or -F functional groups [40]. After etching, the next step is delamination of the multilayer MXene into single or few-layer thick nanosheets. Delamination can be done by the direct sonication of the previously etched MXene, but the yield is quite low in this method. The second and most widely used method for delamination is to intercalate cations or large organic molecules between the layers. The introduction of the appropriate intercalant can increase the interlayer spacing and weaken the interaction between layers. Subsequent mild sonication or shaking can result in the delamination of multilayers into a single nanosheet.

Since HF is a corrosive acid, various other pathways have also been explored where HF can be replaced with any other etchant without compromising the efficiency and yield of the reaction. In this regard, the in situ production of HF by using HCl and a fluoride salt (such as LiF, NaF, and NH_4_F) is the most commonly used synthesis route [37,41,42]. Figure 3 explains the synthesis of Ti_3_C_2_ MXene via the “clay method” and “minimally intensive layer delamination (MILD) method”. Both of these methods involved the in situ production of HF by using LiF/HCl. In the clay method, the end product was a multilayered Ti_3_C_2_ (ML-Ti_3_C_2_), which was obtained by ultrasonication of the bulk-Ti_3_C_2_ MXene, while in the case of the MILD method, a few layered (FL) thick MXene was obtained without further sonication [43].

**Figure 4:**
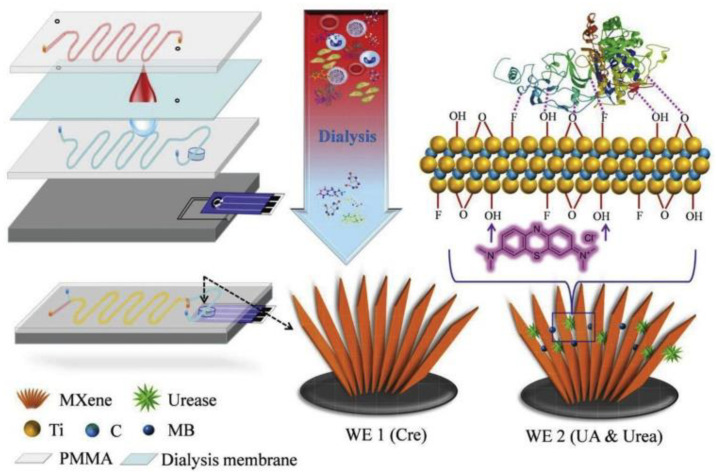
Schematic representation of MXene-based microfluidic chip for the detection of Cre, UA, and urea. Reprinted with permission from [41].

Ti_3_AlC_2_ was the first MAX phase that was transformed into the Ti_3_C_2_ MXene by the etching of the Al layer through concentrated HF. Ti_3_C_2_ MXene has been used extensively as electrode material in biosensing systems. Several superior characteristics make MXene a unique material in sensing systems including hydrophilicity, biocompatibility, large surface area, ease of surface functionalization, and above all efficient electron transport kinetics. Liu et al. reported the development of a MXene-based microfluidic biosensor for multiplexed analysis of biomarkers (i.e., urea, uric acid (UA), and creatinine (Cre)) [41] (Figure 4). The concentration of these biomarkers is an important indicator in patients having severe kidney injuries and those requiring hemodialysis. The developed biosensor chip was based on two MXene modified screen-printed electrodes: one for the detection of UA and urea, and the second electrode for creatinine detection. The electrode for urea and UA analysis was composed of urease/methylene blue/MXene/SPE, while Cre was determined on MXene/SPE. The sensing performance of the multicomponent microfluidic chip was tested in human serum to detect three analytes simultaneously. The sensor showed good sensitivity, stability, and selectivity against all the tested biomarkers. The LOD obtained for UA, urea, and Cre were 5 µM. 0.02 µM, and 1.2 µM respectively. In this design, MXene played dual functions: it facilitated the charge transfer, thus increasing the sensitivity, and secondly, the surface functional groups on the MXene provided an excellent matrix for the immobilization of the enzyme and methylene blue.

Furthermore, MXenes have also been reported for glucose detection. In a recent example, a glassy carbon electrode (GCE) was modified with Ti_3_C_2_ and in the next step, glucose oxidase (GO) was immobilized on Ti_3_C_2_–GCE. The biosensor was tested for glucose analysis. Interestingly, the design facilitated the heterogeneous electron transfer (HET) rate of the Ti_3_C_2_–GC at the GCE surface. It is worth mentioning that when poised at +0.15 V vs. Ag/AgCl reference electrode, the biosensors showed selectivity against common interferences such as ascorbic acid, dopamine, and uric acid, although the concentration was high (0.2 mM each) [42]. In another work, 3-aminopropyltriethoxysilane (APTES) functionalized Ti_3_C_2_–MXene (1 mg·mL^−1^ dispersed in ethanol and water containing 0.1% Nafion^®^) was drop-casted on a GC electrode and the carcinoembryonic antibody monoclonal (anti-CEA) was covalently linked via EDC/NHS chemistry, achieving a label-free and highly sensitive (≈37.9 μA ng^−1^mL cm^−2^ per decade) biosensor for CEA detection [44]. A DNA-based biosensor for the detection of gliotoxin was developed using tetrahedral DNA nanostructures (TDNs)-modified MXene (Ti_3_C_2_) nanosheets, TDNs docking on the MXenes through coordination interactions between the phosphate groups on DNA and titanium, avoiding time consuming and the expensive modification of DNA probes. The biosensing ensemble was entrapped in 0.5% Nafion^®^ and drop-casted on a GCE. The biosensor showed promising results (LoD = 1.63 pg/mL and linear working range between 1.63 and 3260 pg/mL) being able to compete and outperform laboratory HPLC-MS/MS analysis [45]. Another Ti_3_C_2_T_x_-based amperometric biosensor was reported for the sensing of the β-hydroxybutyrate, which is a biomarker for diabetic ketoacidosis. The sensor was composed of a MXene–β-hydroxybutyrate dehydrogenase nanocomposite containing an enzymatic cofactor, the stabilizing protein agent, and a cross-linking reagent. The enzyme/MXene nanohybrid was drop-casted on a gold-printed circuit board modified with 1 mM hexaammineruthenium (III) (Ru(NH_3_)_6_^3+^) as a redox mediator. Operating at −0.35 V vs. Ag/AgCl, the developed biosensor displayed a linear range between 0.36 and 17.9 mM as well as a LOD of 45 µM. The sensor was tested for the determination of β-hydroxybutyrate analyte in (spiked) real serum samples [46]. Moreover, a mediator-free amperometric biosensor was designed for the detection of phenol. The biosensor was composed of MXene as enzyme (tyrosinase) host, chitosan as a binder, and GC as a transducer. The developed Ti_3_C_2_/tyrosinase biosensor enabled the ultrasensitive and rapid detection of phenol. The fabricated biosensor was biased at a low applied potential (−0.04 V vs. Ag/AgCl), which significantly reduces interferences and displayed good analytical performance over a wide linear range (0.05–15.5 µM) with a detection limit (LOD) of 12 nM and a sensitivity of 414.4 mA M^−1^ [47].

### 2.3. Transition Metal Dichalcogenides (TMDs)

TMDs are semiconductors of the type MX_2_, where M is a transition metal atom belonging to group 3 to group 12 of the periodic table (most commonly group IV, V, and VI), while X is a chalcogen (e.g., Te, S, Se) [48,49]. TMDs can be found in different structural phases including trigonal prismatic (2H), distorted octahedral (1T), and dimerized (1T’) coordination of metal ions; the variety in structural phases is attributed to different stacking of the atomic planes formed by the individual layers. TMDs have mixed electronic properties that vary from semiconducting to metallic to superconducting, depending on the structure and composition of the respective TMDs [50,51,52]. Unlike graphene and MXene, TMDs do not exist in a monolayer form. Generally, TMDs possess a sandwich-like structure where a layer of metal atoms is sandwiched between the two hexagonal planes of chalcogens; hence, they are called dichalcogens that collectively form a tri-layered sheet-type structure. The atoms in this tri-layered structure are connected to each other through a strong covalent bond while one tri-layered sheet is connected to its neighboring tri-layered sheet via weak van der Waals forces [53]. The feature that distinguishes TMDs from other NMs is their tunable electronic character that makes it possible to produce TMD in different forms, varying from semiconductor to semimetal, and from true metal to superconductor [54,55]. These conversions become possible by quantum confinement, doping, field effect, and intercalation of various atoms and molecules into the van der Waals layers of TMDs [56]. Most reported TMDs are molybdenum sulfide (MoS_2_), molybdenum selenide (MoSe_2_), tungsten sulfide (WS_2_), tungsten selenide (WSe_2_), and vanadium sulfide (VS_2_). MoS_2_ is the representative TMD, used in many applications, including biosensors [57].

Similar to other NMs, TMDs have also been synthesized by using both top–down and bottom–up approaches. In the top–down approach, bulky layered material is converted to single and few atomic layers thick sheets by means of chemical, mechanical, electrochemical, and liquid exfoliation. Mechanical exfoliation is similar to the scotch-tape method used for graphene synthesis [58,59]. Although this is the fastest method of producing TMDs, the lateral size of the exfoliated single layers is low (typically ≈10 μm), about an order of magnitude smaller than the lateral size of the atomically thin layer graphene obtained by micromechanical exfoliation [60]. The most common TMDs synthesis route is liquid exfoliation, which basically consists of two steps: (i) intercalation and (ii) sonication of bulk material [61,62]. This process is further modified to a more direct approach called sonication-assisted exfoliation or direct liquid exfoliation in a suitable solvent or surfactant [63,64]. This synthesis route is mostly applicable for the large-scale production of MoS_2_ [61]. Chemical and electrochemical exfoliation involves the intercalation of ions such as Li^+^, Na^+^, SO_4_^2−^, etc. These methods are relatively time-consuming but have a high yield of TMDs production with low surface defects and a relatively higher surface area [65,66]. Bottom–up synthesis approaches include chemical vapor deposition (CVD), solvothermal synthesis, and hydrothermal synthesis [67]. Solvo and hydrothermal methods are scalable and readily controlled, while the CVD helps synthesize a specific TMD with a certain thickness [68]. Moreover, the process of direct growth of nanocrystal in CVD is beneficial in avoiding interfacial contamination, which is a common drawback encountered with top–down synthesis methods [68]. Furthermore, the wet ball milling-assisted exfoliation method was used for the synthesis of MoS_2_, delivering multilayers (8 layers) with modest average lateral sizes of 1.54 μm. Studying the efficiency and influence of two organolithium intercalants (i.e., methyllithium-MeLi and *N*-butyllithium-BuLi) on the various TMDs including MoSe_2_, WS_2_, and WSe_2_ c-axis expansion, it was confirmed that the most effective exfoliation process was obtained with BuLi, leading to an efficiency of intercalation in the following order: MoSe_2_ > WS_2_ > WSe_2_, among the investigated TMDs. The use of BuLi showed no significant changes in WS_2_ and WSe_2_ chemical composition, but on MoSe_2_, a consistent increase of Mo(VI) oxidation state and a simultaneous increase in oxygen content with exfoliation were obtained [69]. In addition to these methods, sonication and ball milling-assisted liquid exfoliation, fluid dynamic methods (e.g., vortex fluidic film, pressure-driven fluid dynamics, mixer driven fluid dynamics) have also been used and provide good quality of exfoliated graphene suitable for large-scale use [26].

**Figure 5:**
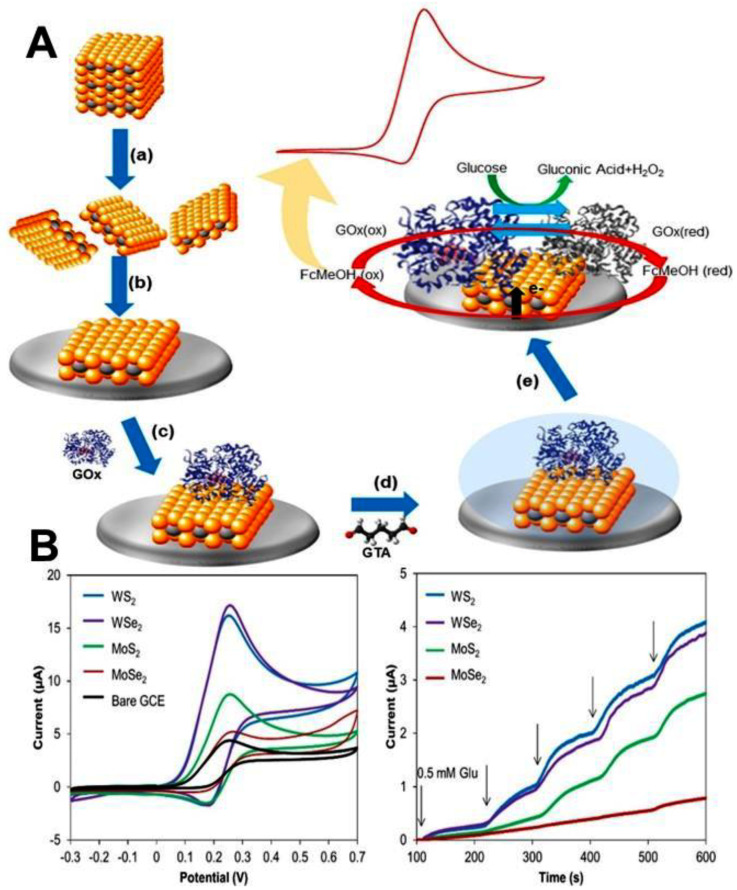
(**A**) Stepwise fabrication strategy of TMDs-based glucose biosensor. (**B**) Voltammetric and amperometric comparison of the analytical performance of different TMDs toward glucose detection. Reprinted by permission from [70].

TMDs have many unique features for electrochemical biosensors, most important of which are their tunable bandgap and efficient heterogeneous electron transfer (ET). Rohaizad et al. presented a comparative study of different TMDs in terms of their performance for glucose detection. To assemble the glucose biosensor, exfoliated WS_2_, WSe_2_, MoS_2_, and MoSe_2_ flakes (dispersed in water) were drop-casted on GCE; then, glucose oxidase (GOx) was immobilized and ultimately cross-linked with glutaraldehyde (as shown in Figure 5). The best analytical performance was obtained with tungsten dichalcogenides (WS_2_, WSe_2_)-based GOx biosensors as compared to their molybdenum counterparts (MoS_2_, MoSe_2_), due to faster ET rate. The ET rate correlated with peak-to-peak separation (ΔEp) in a typical cyclic voltammogram run in 2 mM ferrocene methanol (FeMeOH) in PBS (pH 7) solution. The smallest ΔEp corresponded to the fastest ET rate and vice versa. The ΔEp of four TMDs under study were ranked in the ascending order: WSe_2_ < WS_2_ < MoSe_2_ < MoS_2_ < GC electrode, concluding that the smallest ΔEp was obtained for WSe_2_, which exhibits the fastest ET rate and therefore offers the best electrocatalytic performance for biosensing applications [70] (Figure 5).

### 2.4. Hybrid Hierarchical Assemblies Based on Combined Heterogeneous Nanostructures

Many biosensors have been developed by harnessing the synergistic properties of different types of nanomaterials, which is a strategy that has been shown to enhance performance. Hybrid nanomaterials are made of metals NPs (e.g., Au, Ag, and Pt) and metal oxides (ZnO, TiO_2_, CeO_2_, etc.). NPs decorated on the surface and in between the layers of 2D nanosheets are the most reported in the literature [71,72,73]. These hybrid composites have shown higher conductivities and catalytic properties and faster charge transfer kinetics as compared to that of their parent constituents. These hierarchical structures can be classified into two categories: as structures with geometrical complexities containing nanoscale building blocks and as structures composed of multi-components. The geometrical complexities in different dimensions lead to enhancement in high surface area, photocatalytic activity, and high scale order arrangement for electronic applications. The combination of multiple components and complex geometries results in enhanced properties for applications such as photocatalytic water treatment, environmental biosensing, gas sensing, and monitoring. A complete and detailed understanding of hybrid nanostructures and their behavior in biological environments is needed to further develop and take advantage of their full potential for biosensing applications [74,75].

Sun et al. demonstrated the formation of CuO hierarchical nanoflowers obtained by in situ dissolution–precipitation and its use as an electrode material for non-enzymatic glucose biosensors [76]. The growth of metal oxide nanostructures is controlled by the water-dependent precursor transformation phenomenon. Cheng et al. [77] fabricated hierarchical core–shell Co_3_O_4_/CuO nanorod arrays supported on carbon cloth as materials to construct a non-enzymatic glucose sensor with high sensitivity and good selectivity. The sensor exhibited a sensitivity of 5450 µA mM^−1^ cm^−2^ with a fast response time. Tran and Kim proposed the use of organic–inorganic hybrid nanoflowers as a multifunctional hierarchical nanostructure for biosensing [78].

**Figure 6:**
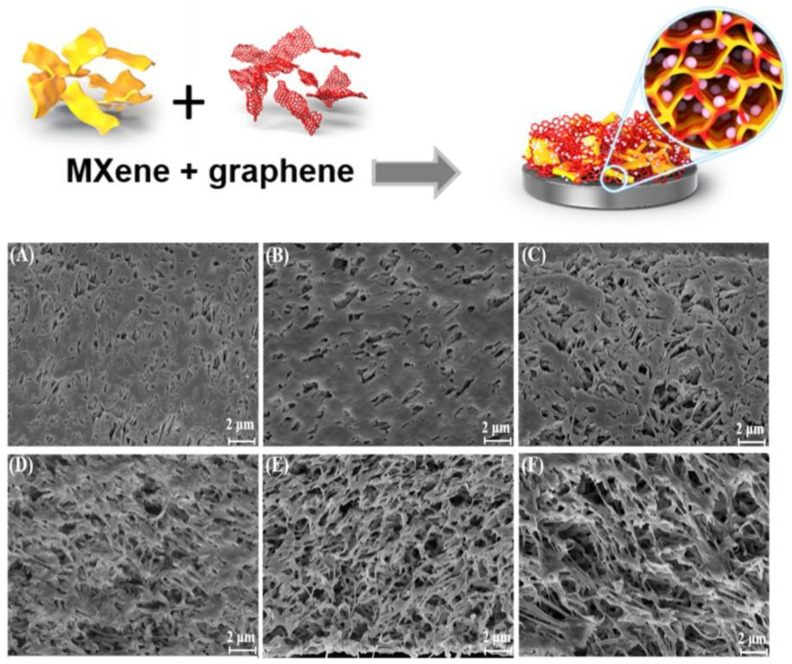
Graphical representation of the MG porous structure. SEM images of (**A**) Ti_3_C_2_T*_x_* (**B**) graphene (**C**) MG (2:1) hybrid film, (**D**) MG (1:1) hybrid film, (**E**) MG (1:2) hybrid film, and (**F**) MG (1:3) hybrid film. Reprinted with permission from [79].

In other reports, the use of biocompatible TiO_2_ nanoparticles provided a microenvironment enabling preservation of the bioactivity of immobilized biomolecules and retaining their stability for long time use, thus improving the shelf life of the biosensor [76,79,80]. Various TiO_2_/2D nanocomposites have also been reported [81,82]. Wu et al. reported a 3D porous MXene–graphene (MG) nanocomposite for the development of a glucose biosensor. MXene contains a rich unsaturated surface with unpaired electrons and abundant functional groups (e.g., -O, -OH, or/and -F groups) without burdening the metallic conductivity [83]. The MG hybrid combined the mechanical strength and hydrophilicity of MXene with the strikingly high conductivity of graphene. The resulting nanohybrid possesses a 3D porous morphology in which the size of internal pores was tuned by simply changing the MXene to graphene ratio. The porous structure was reported to maximize the biomolecule loading capacity of the biosensor and also enhanced the redox performance of the biosensor [79]. The graphical representation and SEM images of MG hybrid are shown in Figure 6.

**Figure 7:**
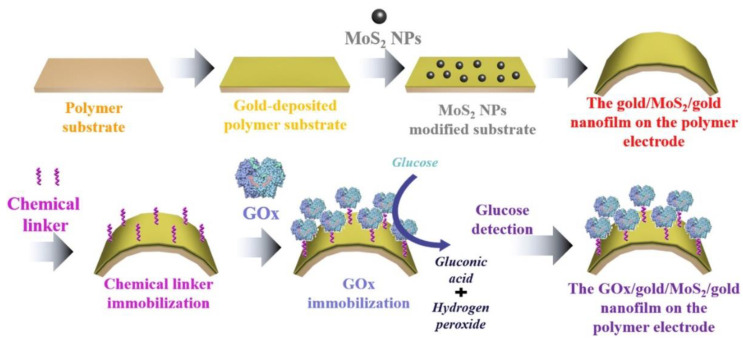
Stepwise details of the fabrication process of a wearable glucose biosensor composed of a GOx/gold/MoS_2_/gold nanofilm on a flexible polymer substrate (reproduced with permission from [80]).

Likewise, Yoon and co-workers reported a flexible/wearable glucose biosensor by immobilizing GOx on a flexible polymer substrate modified with MoS_2_/gold nanofilm [80]. The fabrication process involved the sputtering of Au on a commercially available polymer electrode followed by the spin coating of MoS_2_ NPs, and finally, another layer of Au was sputtered on the top of MoS_2_. The GOx was immobilized via a chemical linker. The developed biosensor showed satisfactory analytical performance with a limit of detection of 10 nM. The enhanced sensitivity was attributed to the synergistic effects of MoS_2_ and gold layers that resulted in fast charge transfer kinetics. The micro-fatigue test data revealed that the flexure extension of this biosensor (i.e., 3.48 mm) was much higher than that of a gold-coated silicon-based sensor that possesses a flexure extension value of 0.09 mm. The systematic fabrication strategy of the developed biosensor is shown in Figure 7.

## 3. Role of 2D Nanomaterials in Electrochemical Sensing Platforms

### 3.1. Improvement in Electron Transfer Kinetics with 2D Nanomaterials

Carbon materials are some of the most used electrode materials for electrochemical biosensors. A typical procedure involves the modification of carbon electrode substrates such as GC or screen-printed carbon electrodes (SPCE), as well as other types of electrodes providing high sensitivity and fast redox transfer. Two-dimensional (2D) carbons are known to improve the heterogeneous electron transfer rate constant (k°) [84] of the developed electroanalytical devices; k° best describes both the nature of the redox couple involved and the electrode material. It is worth mentioning that the nature, size, and dimensionality of the material and its synthesis/isolation have an impact on electron transfer kinetics. Comparing k° (cm s^−1^) of graphene_ME_ (mechanically exfoliated), graphene_CVD_ (chemical vapor deposited), and graphite_BP_ (basal plane), it was observed that heterogeneous electron transfer rates follow the order: graphene_ME_ (0.5 cm s^−1^) > graphene_CVD_ (4.2 × 10^−2^ cm s^−1^) > graphite_BP_ (7 × 10^−3^ cm s^−1^) [85]. Investigating other allotropes of carbon, such as GDY, graphene, and multiwalled carbon nanotubes (MWCNTs), the heterogeneous electron transfer rates constants for an outer sphere redox mediator (i.e., hexaammineruthenium (III)) were the following: k°_GDY_ = 0.030 < k°_G_ = 0.035 < k°_MWCNTs_ = 0.12 cm s^−1^ [86].

Similarly, comparing the k° of bulk (*b*MoSe_2_) and exfoliated (*e*MoSe_2_) molybdenum selenide or bulk (*b*WS_2_) and exfoliated (*e*WS_2_) tungsten disulfide, it is observed that heterogeneous electron transfer rates are improved one order of magnitude with respect to the bulk of the crystal: k°*_b_*_MoSe2_ (2.70 × 10^–5^ cm s^−1^) < k°*_e_*_MoSe2_ (9.17 × 10^–4^ cm s^−1^) and k°*_b_*_WS2_ (3.40 × 10^–7^ cm s^−1^) < k°*_e_*_WS2_ (2.75 × 10^–6^ cm s^−1^). On the contrary, for bulk molybdenum disulfide (*b*MoS_2_), the k°*_b_*_MoS2_ (2.11 × 10^–3^ cm s^−1^) > k°*_e_*_MoS2_ (2.26 × 10^–4^ cm s^−1^) of exfoliated (*e*MoS_2_) molybdenum disulfide. Similarly it was observed for tungsten diselenide (WSe_2_), k°*_b_*_WSe2_ (5.48 × 10^–5^ cm s^−1^) > k°*_e_*_WSe2_ (1.21 × 10^–5^ cm s^−1^). As a benchmark, the k° values of glassy carbon_E_ (electrode) and Pt_E_ (electrode) are 2.78 × 10^–4^ cm s^−1^ and 4.12 × 10^–3^ cm s^−1^, respectively [69]. As observed for ML-Ti_3_C_2_T_x_ and FL-Ti_3_C_2_T_x_ the k° value increases on moving from ML-Ti_3_C_2_T_x_ (*k*° = 0.09533 cm s^−1^) to FL-Ti_3_C_2_T_x_ (*k*° = 0.00503 cm s^−1^), which agrees with most of the literature on other 2-dimensional (2D) materials such as graphene and TMDs [43].

### 3.2. 2D Nanostructures as Electrode Material Modifier

The main utilization of 2D nanomaterials is as electrode modifiers, providing large area support for the immobilization of biomolecules. Their surfaces can be functionalized with linkers and biomolecules, providing in addition to anchoring points and improved conductivity, mechanical stability, and direct electron transfer. Graphene was often mixed with AuNPs or electrodeposited polymers such as polypyrrole in order to improve the mechanical stability, the electroactive area, and the current intensity/sensitivity. In addition to composite coatings made by drop-casting mixtures of nanomaterials, linkers, and polymers, electrophoretic deposition emerged as a versatile method for the deposition of graphene and nanocomposite coatings with the increased active area, conductivity, and functional groups for anchoring biomolecules. As an example, Srivastava et al. developed biosensors for Aflatoxin B1 where specific anti-Aflatoxin B1 antibodies were immobilized on ITO electrodes coated with rGO, rG/NiNPs, and rGO/AuNPs composites by electrophoretic deposition. Modification of ITO electrodes by including metallic nanoparticles such as Au or Ni in the rGO coating leads to higher conductivity and increased active area. This was translated into larger heterogeneous electron transfer rate constants (i.e., faster kinetics of the electron transfer), enhanced sensitivity, and wider detection range compared to sensors lacking metallic nanoparticles [87,88,89]. More details on the integration of heterogeneous nanostructures with biomolecules are provided in Section 4 of this review.

### 3.3. 2D Nanostructures as an Electrochemically Active Label and Support for Electroactive Probes

Graphene-oxide nanoplatelets were bound by π–π interactions to an aptamer for OTA attached to the electrode surface. The electrochemical reduction of nanoplatelets produces a cathodic current that is used as the analytical signal [90]. An rGO solution was added to an aptamer biosensor after incubation with the sample. RGO attached to the aptamer and detection was done by DPV using [Fe(CN)_6_]^4−/3−^ as a redox probe [91]. Nanocomposites of MoS_2_ nanosheets/AuNP were used in a similar manner, for the signal amplification in an aptasensor for OTA [92].

**Figure 8:**
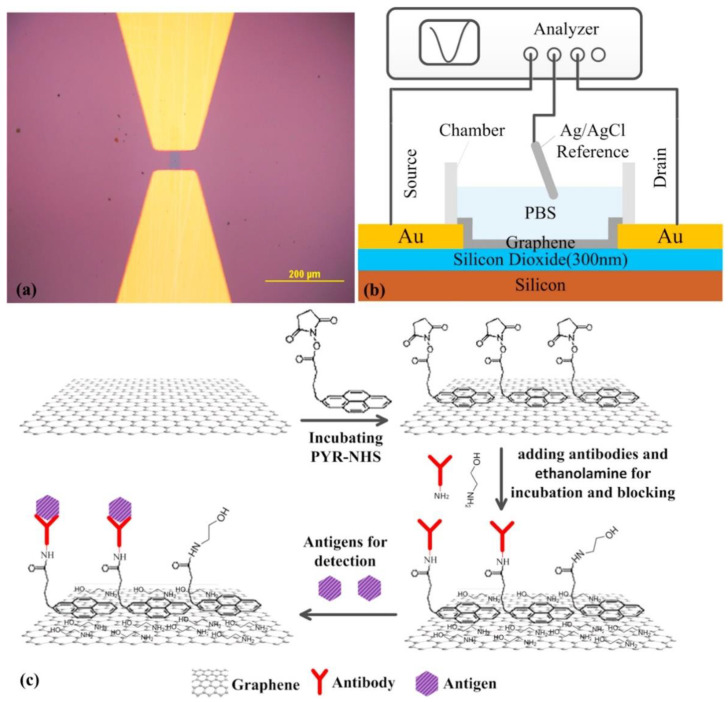
(**a**) Optical micrograph of the graphene channel; (**b**) Schematic diagram of solution gated GFET biosensor; (**c**) The schematic diagram of all the modification steps for GFET (reproduced with permission from ref [93]).

One of the main applications of hierarchical nanostructures is in the development of field-effect transistor (FET)-based biosensors for the label-free detection of various analytes. The variations in channel conductance upon biomolecular interaction resulted in changing the drain current level that consequently affects the repeatability of signal in FETs. Therefore, it always remained a challenge to get a steady-state reproducible signal in an FET-based biosensor. To overcome this obstacle, the sensing channel was patterned with 2D materials. Single-layer graphene, obtained via chemical vapor deposition (CVD) was transferred, etched, annealed, and electrically contacted in order to fabricate a graphene field-effect transistor (GFET), the graphene-based channel being afterward altered by the immobilization of antibodies targeting carcinoembryonic antigen (anti-CEA), thus delivering a label-free immunosensor [93]. The GFET-based immunosensor (Figure 8) reported a limit of detection (LOD) of less than 100 pg mL^−1^, which is much smaller than the cut-off value (5 ng mL^−1^) in clinical diagnosis. Moreover, a MoS_2_ FET-based biosensor for the ultrasensitive detection of DNA by employing phosphorodiamidate morpholino oligos (PMO)–DNA probes was recently reported. The MoS_2_ channel was simply obtained via drop-casting the negatively charged MoS_2_ suspension onto the APTES functionalized sensing channel and removing the excess of MoS_2_ flakes by ultrasound and thoroughly washing with DI water. It is worth mentioning that the proposed biosensor was able to specifically discriminate the complementary DNA from one-base mismatched DNA, three-base mismatched DNA, and non-complementary DNA, thus being able to be used for assessing and screening single nucleotide polymorphism (SNP) [94]. A highly sensitive (2.87 × 10^5^ A/A for 10 mM glucose at V_G_ = 20 V) and reusable tungsten diselenide (WSe_2_) FET was modified with GOx, and a glucose FET-based biosensor was achieved. Mechanically exfoliated WSe_2_ flakes were treated using a weak power O_2_ plasma to promote chemical functionalization with APTES before immobilizing glucose oxidase via glutaraldehyde crosslinking [95].

The majority of biosensors that incorporate 2D nanomaterials, discussed in detail in Section 4, were produced by modifying solid electrodes (mostly GCE, Au, ITP, and Pt) by drop-casting nanomaterial modifiers or nanomaterial composites. A small number of biosensors were produced by methods that can ensure a higher degree of control of layer thickness and homogeneity, e.g., electrophoretic deposition [88]. Despite the fact that the fabrication process of some devices includes several sequential drop-casting steps, the reproducibility of all biosensors was in general good, with reported RSD < 10% even for sensors from different batches.

## 4. Integration of Biomolecules with 2D Nanostructures

**Figure 9:**
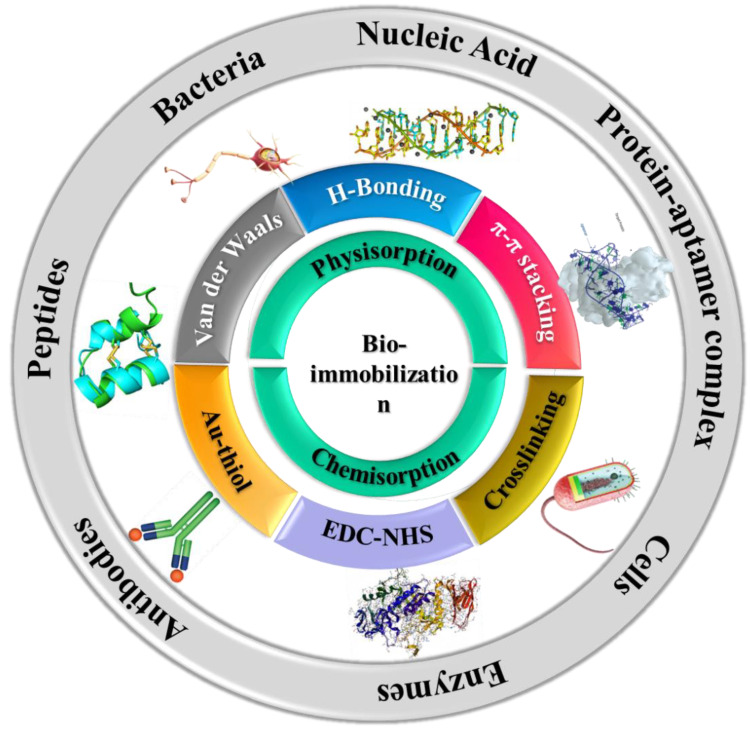
Commonly used immobilization strategies of biological molecules onto 2D nanomaterials and layered structures.

Figure 9 summarizes some commonly used immobilization strategies for the deposition of biomolecules onto the surface of nanomaterials, as discussed in the following sections.

### 4.1. Bacteriophages

In a biosensor for the detection of *Staphylococcus arlettae*, bacteriophages were covalently immobilized on the surface of graphene-modified SPE through carbodiimide chemistry. The graphene-modified SPE was pre-treated by electrochemical oxidation at +1.0 V to maximize the number of carboxyl groups on their surface [96].

### 4.2. Nucleic Acids

Nucleic acids were immobilized on 2D nanomaterials by various non-covalent and covalent methods, including (i) chemisorption of thiolated aptamers to AuNPs [97,98,99,100,101]; (ii) hybridization with complementary capture DNA sequence fixed on electrode surface [102,103,104]; (iii) by biotinin–avidin affinity, e.g., a biotinilated aptamer attached by affinity to strepravidin-modified graphene on an ITO electrode[105]; (iv) by host–guest interaction with β-cyclodextrin (β-CD); (v) via coordinative linking between a phosphate terminal group in a DNA sequence and Zr-OH group in metal organic frameworks MOF [106]; (vi) adsorption, e.g., to graphene, [107] or to BiOBr nanoflakes/*n*-doped graphene nanocomposite by π–π stacking [108]; (vii) cross-linking with glutaraldehyde between an aptamer with an amine-terminal group and nanomaterial functionalized with linkers containing amine groups, e.g., an aptamer for the cyanobacterial toxin cylindrospermopsin was bound to a nanocomposite of graphene-thionine [109]; (viii) covalent attachment via carbodiimide chemistry to carboxyl groups present in the nanomaterial itself or in linkers attached to the nanomaterial: e.g., on carboxylated graphene oxide-modified screen printed electrode, directly [110] or via a spacer [111], on carboxylated polystyrene nanospheres previously fixed on graphene via layer by layer modification with poly(diallyl dimethylammonium chloride) [112], etc.

### 4.3. Antibodies

Antibodies were either physically adsorbed (e.g., on composites such as PtNP-CoTPP-rGO [113]) or covalently immobilized on the 2D nanomaterials or their composites. A typical example of chemisorption is linking amine terminal antibody to carboxyl functional groups in oxidized graphene [114]. Another example includes the chemisorption of antibodies on a carboxyphenyl film formed by electrochemical reduction of in situ generated aryl diazonium salt on graphene-modified SPE [115].

### 4.4. Peptides

A biosensor for the detection of Botulinum neurotoxin A was developed by the covalent attachment of the SNAP−25-GFP peptide on rGO at the electrode surface via a pyrene-butyric acid linker [116]. The peptide represents an enzymatic substrate for the Botulinum neurotoxin, and the biosensor operated on the principle of measuring the protease activity of the toxin.

### 4.5. Enzymes and Proteins

Nanomaterials of different morphologies, compositions, and functional groups were frequently used as immobilization matrices in biosensors, all affecting the characteristics of the immobilized biomolecules. Once immobilized on the surface, both the catalytic efficiency of an enzyme and the sensitivity to inhibitors can be affected [117]. Generally, simple strategies are used for anchoring enzymes onto the nanostructures such as physical adsorption in which the enzymes are deposited by drop-casting on the nanomaterial-modified transducer or mixed with nanomaterials along with a binder to obtain a homogeneous dispersion prior to the deposition on the electrode. The immobilization of enzymes on or within 2D nanomaterials has been reported to facilitate the direct DET from enzymes to electrodes and improve the affinity of the enzymes for their substrate. The direct immobilization of the enzyme on the electrode has shown lower sensitivities. For example, the sensitivity toward Bisphenol A (BPA) of a biosensor that was fabricated by the adsorption of tyrosinase on GDY was 2990.8 mA cm^−2^ M^−1^, double that in the absence of GDY [35]. Tyrosinase adsorbed on hydrophilic graphene leads to better sensitivity (3108.4 mA cm^−2^ M^−1^) than a similar sensor with MWCNT (1557.3 mA cm^−2^ M^−1^) or one without using any nanomaterial (1026.6 mA cm^−2^ M^−1^) [118].

An improvement in the catalytic activity of GOX was reported for a biosensor where GOx was adsorbed on Nafion/Au/Ti_3_C_2_T_x_ MXene nanocomposite at the surface of a GCE. The better performance was attributed to the combined high conductivity of AuNPs and the good in-plane conductivity of MXene nanosheets [119]. Furthermore, cytochrome C (Cyt C) was adsorbed in the mesoporous channels of zeolitic imidazolate framework−8 (ZIF−8) and immobilized on the electrode with ABTS and Nafion [106]. The Michaelis constant K_m_ of the immobilized Cyt C was about half of the value for the free enzymes in the solution. In another study, catalase was adsorbed on boron nitride sheets dispersed in chitosan and deposited at the surface of a GCE [120]. This approach enabled the DET of catalase for the detection of the plant hormone forchlorfenuron based on enzyme inhibition. The biosensor was included in a Flow Injection Analysis (FIA) system enabling the detection of forchlorfenuron in the range from 0.5 to 10.0 µM. Further improvements in biosensor performance appear necessary when comparing the detection range with the current maximum residue limit detection (MRL) of 0.04 µM [120]. In a similar example, nanoflakes of black phosphorous (BP) prepared by water-phase exfoliation were functionalized with poly-l-lysine (pLL), and the hybrid material was drop casted on a GCE. Hemoglobin was deposited on the modified electrode and then covered with a film of Nafion. The DET from hemoglobin to the electrode was improved on the pLL-BP-GCE electrode [121].

Mixing enzymes with chitosan and drop-casting the mixture on the electrode is one of the most widely used electrode functionalization strategies. A GCE modified with GOx/chitosan/NH_2_-MIL−125(Ti)/TiO_2_ (MIL stands for Materials from Institute Lavoisier) was used to produce a photo-electrochemical sensor for acetochlor based on enzyme inhibition [122]. Another most common approach for bioimmobilization is crosslinking the surface of the electrode with suitable chemistry. For example, HRP was immobilized and entrapped in the phosphorene film by crosslinking with glutaraldehyde [123]. In another report, electrostatic interaction was between positively charged HRP, and a negatively charged composite of MoS_2_–graphene was utilized for the adsorption of biomolecule [124]. Likewise, laccase was immobilized on graphene quantum dots by electrostatic interactions [125]. Xanthine oxidase (XOD) was adsorbed on a GCE electrode modified with a nanocomposite of graphene and TiO_2_, followed by coating with a layer of Nafion [126]. Acetylcholine esterase (AChE) was immobilized by cross-linking with glutaraldehyde in a matrix of BSA on top of a TMD coating to obtain a biosensor for fenitrothion [127].

## 5. Biosensing Applications of 2D Nanomaterials for Food and Environmental Analysis

Table 1, Table 2, Table 3 and Table 4 summarize the various types of biosensors that incorporate 2D nanostructures reported in the literature for detection of viruses, pathogens, and bacterial toxins (Table 1), mycotoxins (Table 2), marine toxins (Table 3), and other targets, including phenolic compounds and allergens. The examples summarized in Table 1 reflect various approaches for the detection of virus, bacteria, and bacterial toxins by direct, competitive, and sandwich-type tests, using antibodies, aptamers, bacteriophages, or peptides as biorecognition elements. The role of the 2D nanomaterials including graphene, MoS_2_, and MOF was mainly as large-area support facilitating the efficient immobilization of the biorecognition elements. To enhance the sensitivity of the assay, the electrical conductivity of the sensor was improved by using nanomaterial composites, typically with Au nanostructures [128,129], or by using enzyme-based signal amplification systems [106,130]. Meanwhile, the preferred detection methods were DPV and EIS with [Fe(CN)_6_]^4−/3−^ as a redox probe. Alternative voltammetric methods were also noted, e.g., based on the stripping of Ag [130], detection of hematoxylin as a DNA intercalator [129], or the detection of ferrocene as the electroactive label of the detection antibody in a sandwich-type assay [128]. The complexity of the test was reflected in the time per assay. Remarkably, a very short analysis time, including a 2 min incubation with the sample at 37 °C, was reported for an impedimetric biosensor for *Staphylococcus arlettae* [96]. The biosensor consisted of a bacteriophage-modified graphene-screen-printed electrode and despite the short incubation time, its limit of detection for *Staphylococcus arlettae* was impressive: 5 cfu mL^−1^. Moreover, the biosensor’s feasibility was tested with spiked water and apple juice samples.

Notably, a more complex biosensor did not always provide a greater sensitivity of detection. In many cases, a simpler design can provide more robust devices due to the ability to control a fewer number of variables. For example, in a competitive test for the detection of *Salmonella Typhimurium*, the specific aptamer included in a nanocomposite with AuNPs and HRP was incubated with the sample [106]. The excess, unbound aptamer was hybridized to a complementary DNA sequence and fixed at the surface of an electrode coated with a MOF–graphene composite layer. The attachment of the DNA sequence with a phosphate group at its 5′-end to the electrode was achieved by the coordinative binding between the phosphate and the Zr-OH groups of the MOF. The determination of *Salmonella Typhimurium* was based on measuring the amount of unbound aptamer by using the catalytic activity of the HRP–aptamer–AuNPs composite captured at the electrode surface. This was done by measuring the intensity of the cathodic current produced at the electrode surface following the reaction of H_2_O_2_ and hydroquinone, which was added in the electrolyte. The total analysis time with this biosensor was 3 h, and the linear range reported was 2 × 10^1^–2 × 10^8^ cfu mL^−1^. For comparison, a simpler biosensor based on direct detection and classic measurement by DPV using the [Fe(CN)_6_]^4−/3−^ probe was reported to achieve measurements of *Salmonella Typhimurium* in ten minutes with a similar detection range. In this second biosensor, the aptamer was immobilized by adsorption on a GCE modified with a composite of reduced graphene oxide and multiwalled carbon nanotubes. From the data presented, it cannot be inferred how the non-specific adsorption in real samples of complex matrices would be prevented with this biosensor, since no particular coating or washing procedure to prevent fouling was reported. Nonetheless, an application for measurements in raw chicken meat was described, and a general agreement was claimed between biosensor results and a classic cell culture method; i.e., *Salmonella Typhimurium* was detected in three samples but was not detected in the other two samples. It is important to note also the more in-depth selectivity study by Appaturi et al. [131] including, in addition to five types of bacteria, other serovars of *Salmonella enterica*. While the biosensor was not responsive toward *E. coli, K. pneumonia*, *P*. *aeruginosa*, *S. aureus*, and *Enterococcus faecalis*, it measured other serovars of *Salmonella enterica* with similar sensitivity as *Salmonella Typhimurium*. This is due to the particularities of the aptamer selection process. Since the same aptamer was used in both biosensors discussed above, biosensor developers should be cautioned about the intrinsic limitations of the selected recognition element.

Another example of a bioanalytical device enabling the detection of Salmonella Typhimurium in less than 30 min is an impedimetric immunosensor where specific antibodies were covalently attached to “porous graphene” at the electrode surface [132]. The multilayer coating with a thickness of 15–20 µm and elemental composition of 97.5% C and 2.5% O was obtained by laser induction from polyimide. The electroactive surface area of the coated electrode was 50% larger than the geometric one. Nonetheless, as expected from the elemental composition and the presence of defects, the charge transfer kinetics for the redox ferro/ferricyanide couple was slower than for CVD graphene; for example, the peak separation was larger than 166 mV, and the heterogeneous electron transfer rate constant was k_0_ = 0.0146 cm s^−1^. To prevent non-specific adsorption, the biosensor surface was passivated with a commercial Superblock blocking buffer. When tested with chicken broth samples, the sensitivity of the immunosensor was about half of the value recorded in buffer solutions (i.e., 24 Ω log cfu^−1^ mL compared to 42 Ω log cfu^−1^ mL), indicating a significant matrix effect that should be addressed by appropriate calibration procedures. The detection limits in buffer and chicken broth were similar, 13 cfu mL^−1^ in chicken broth and 10 cfu mL^−1^ in buffer, respectively [132].

A nice illustration of the different analytical possibilities obtained by coupling 2D nanomaterials with biomolecules is offered by two biosensors for the detection of Botulinum neurotoxin A. Botulinum neurotoxin A lightchain is the most toxic of the seven serotypes of Botulinum neurotoxin produced by the foodborne pathogen *Clostridium Botulinum*. A Au electrode was modified with chemically reduced GO by drop casting, and the synaptosomal-associated protein 25 (SNAP−25) peptide was covalently immobilized on this surface by carbodiimide chemistry via a pyrene-butyric acid linker [116]. SNAP−25 is an enzymatic substrate for Botulinum neurotoxin A lightchain. Upon incubating the biosensor with samples containing the toxin, due to the protease activity of the toxin lightchain, the peptide was cleaved. This lessened the electrostatic repulsion and the steric barrier effects observed with the ferri/ferrocyanide redox couple. The increase in the intensity of peak current measured by DPV was proportional to the amount of toxin in solution; moreover, heat-denatured toxin A or toxin B did not interfere due to lack of protease activity. In an alternative, a rather classical approach, a GCE was modified with a composite of AuNPs–graphene–chitosan, and a specific antibody was covalently attached to a self-assembled monolayer of mercaptopropionic acid at the surface of the AuNPs [133]. Non-specific adsorption was prevented by blocking the non-functionalized sites at the electrode surface with BSA. This biosensor achieved a detection limit of 0.11 pg mL^−1^ Botulinum neurotoxin A, while the peptide-based biosensor reached a detection limit of 8.6 pg mL^−1^. Both devices were applied for the analysis of spiked milk. For the immunosensor, recoveries of 102.4–103.8% for spiked milk indicate a good accuracy [133]. In the peptide-based biosensor, washing with a solution containing Tween 20 after the incubation with spiked skimmed milk was effective to remove non-specific adsorption. The matrix effect seems negligible, based on the similarity of peak current changes recorded for samples spiked at three concentrations higher than the DL, i.e., 10, 50, and 100 pg mL^−1^, compared to those recorded for the same concentrations in buffer solution [116]. Unfortunately, the stability data and the length of the incubation time with the sample were not reported for the peptide-based biosensor, while the storage stability of the immunosensor is limited to 4 days at 4 °C. The incomplete characterization is a typical occurrence in biosensor literature and a major hurdle when comparing the performance of different concepts and devices.

## 6. 2D-Based Biosensors for Wearable Devices

An emerging area of growth that can benefit from the implementation of 2D nanostructures is the development of portable and wearable biosensing devices in healthcare. These innovative platforms involve the integration of the biosensing components with wearables and require specific designs to achieve flexibility and wearability to the user for attaining health monitoring. The development of wearable and flexible bioelectronic devices is one of the most exciting and promising directions in the biosensing field. These devices are designed with the goal of complementing the capabilities of existing wearable sensors that are currently limited to tracking physical activities and vital signs. Significant effort is dedicated to developing devices that can monitor disease biomarkers, for example, for non-invasive monitoring glucose and lactate levels in sweat.

**Figure 10:**
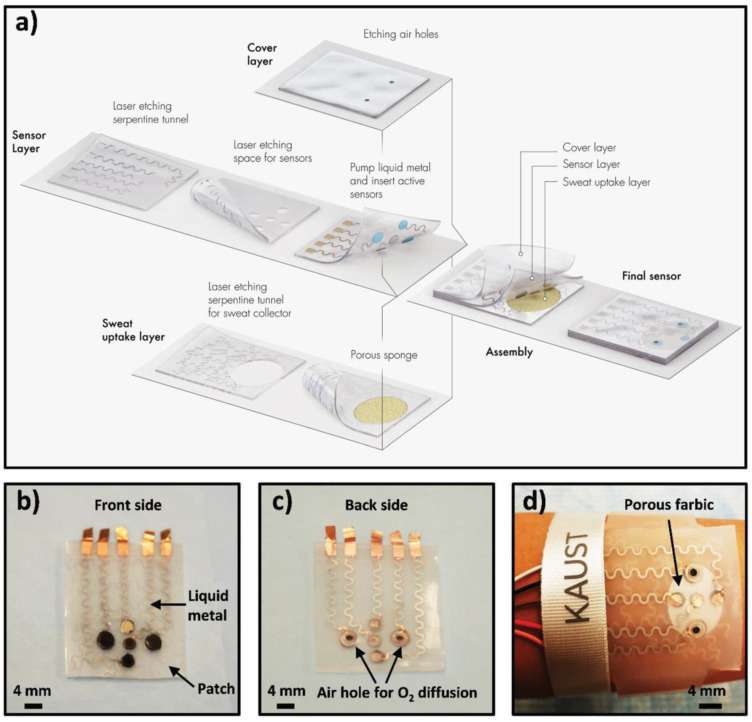
Schematic drawings and corresponding images of the wearable sweat sensor (**a**) components of the sensing system including a sweat-uptake layer, a sensor layer, and a cover layer. (**b**) Front side of the patch, (**c**) Back side of the sensor, (**d**) Images of the sensor wristband laminated on human skin. Reprinted with permission from [125].

Due to their electronic and mechanical properties, 2D materials have the potential to serve as platforms for creating bending and flexible bioelectronic sensors that can be attached to skin or textiles. The recent advancements in nanopatterning and printing can facilitate the manufacturing of inexpensive components that integrate 2D nanomaterials and interface them with biomolecules for wearables. Recently, a MXene-based wearable biosensor was reported for the multi-component analysis of human sweat [168]. The developed flexible biosensor patch can be worn on the wrist with replaceable sensors. The biosensor was able to monitor the pH, glucose, and lactate levels in sweat. The replacement of the sensor is an effective strategy to prolong the service life of the biosensor patch and to deal with the unavoidable enzyme inactivation problem. Figure 10 provides an overview of the fabrication and working principle of the developed sensor patch. As shown in the figure, the patch has a flexible skin conforming design in which all three sensors (pH, glucose, and lactate) were placed in separate, replaceable compartments. Therefore, once any of the three sensors stopped working, it can easily be replaced with a new chip. The sensing chip is based on a Ti_3_C_2_ MXene Prussian blue (PB) composite with and the corresponding enzyme (GOx or lactase). The performance of this sensor was demonstrated by analyzing the level of glucose and lactate in artificial sweat. Later, the sensor patch was further tested on human subjects. The sensor displayed satisfactory performance with high selectivity for the targeted analytes. The idea behind the sensing patch has the potential to generate marketable products that can be used for the noninvasive detection of biomarkers at early stages.

## 7. Conclusions and Future Outlook

In summary, 2D materials and hybrid configurations show potential as candidate platforms for the development of next-generation electrochemical biosensing devices, including flexible and wearable user-interactive sensors. This paper provided an overview of the 2D nanostructures, their properties, and their applicability as electrode materials, modifiers, and support for the immobilization of biomolecules. Several examples of applications in the food, clinical, and environmental fields have been discussed and their performances summarized in extensive application-related tables. As compared to previously developed nanomaterials, engineered 2D nanostructures provide some advanced capabilities such as a single-layered or stacked layered structure providing a very large surface area, organized and tailorable morphology, flexibility in design, and superior conductivity. Their surface can be decorated with other materials, such as Au or Pt NPs, and their terminal groups can be easily functionalized with biomolecules, creating hybrid structures with the enhanced electrical, catalytic, or optical performance and detection capabilities for targeted biomolecular recognition. These physicochemical properties of 2D nanostructures make them a suitable candidate for biosensing, particularly for the rapidly growing flexible and wearable devices.

In addition to the properties of 2D nanomaterials discussed here, there are still some challenges that need to be addressed to exploit their full potential and achieve the practical realization of biosensing devices. Most demonstration of sensing capabilities of the 2D nanomaterials is still restricted to lab-based research. In order to move this concept to the market, the simple integration of the hierarchical materials with the biomolecules and measuring of the signal is not sufficient. Following the initial concept, there is a need to further develop and integrate the biosensing system with electronic circuitry and data analysis tools, deploy, and fully validate these devices in realistic environments. The scalable fabrication and large-scale manufacturing of these devices also require developing methods that will enable the automatic production of these nano assemblies and their deposition on flexible supports through approaches such as printing. In addition to the deposition of the 2D nanostructure or the in situ growth of the nanostructure directly on the sensing platform, efforts should be dedicated to the immobilization of the bioreceptor, e.g., enzymes onto the printed layers to incorporate both the transducer and the biomolecule components for biorecognition. Improvements in manufacturing and device fabrication are needed to lower fabrication costs, improve reproducibility, and facilitate large-scale production of units, which are all essential steps toward commercialization. In addition, issues related to connectivity with smartphones, Bluetooth, and other widely used communication devices, and data transmission features are needed to develop a user-interactive communication and data analysis. Further, special attention should be paid to the mechanical deformation of the wearable sensors and how this affects the performance over time. Moreover, such systems still need to be studied to address the consideration of strain tolerance of not only the 2D nanomaterial but their interactions with the surrounding environment. Second to the structural modifications, the environmental stability of the developed 2D materials and methodologies to mitigate their degradability must be taken into account. Third, developing structures with predictable and rationally designed characteristics should be sought rather than pursuing exploratory trials to find the ideal configurations. For example, how the properties of 2D nanomaterials affect performance of the sensing device should be systematically studied, and a structure–property relationship should be developed through combined theoretical and experimental investigations. Such developments require an integrated interdisciplinary approach and close collaborations of analytical chemists, materials scientists, biochemists, physicists, electrical engineering experts, and practitioners in the fields of use. These efforts can lead to portable diagnostic devices that are particularly useful for field analysis and widely distributed low-cost screening.

## Figures and Tables

**Table 1:** Examples of biosensors including 2D nanomaterials for the detection of viruses, pathogens, and bacterial toxins.

Analyte	Description/Immobilization Details	Performances	Selectivity	Real Sample Application	Ref
*Porcine epidemic diarrhea virus (PEDV)*	Ab/AuNP-MoS2-rGO/GCE. Detection by faradaic EIS	Range: 82.5–1.65 × 10^4^ TCID_50_ mL^−1^. Incubation time with the virus: 140 min at 37.5 °C. Stability: 89.2% of activity after 2 months at 4 °C.	No interferences from *Salmonella* spp., *Campylobacter jejuni*, and *E. coli*	Spiked pig manure	[134]
*Salmonella typhimurium*	cDNA –MOF (type UiO−67) –Gr/GCE and APT-AuNP-HRP for signal amplification. Detection by DPV	Range: 2 × 10^1^–2 × 10^8^ cfu mL^−1^; DL: 5 cfu mL^−1^; Analysis time: 3 h; Stability: 91.3% of activity after 2 weeks at 4 °C	*S. aureus, S. flexneri, E. coli*, and *Streptococcus* not interfering	Spiked milk	[106]
*Staphylococcus arlettae*	Bacteriophages/Gr electrodes. Detection by faradaic EIS	Range: 2.0–2.0 × 10^6^ cfu mL^−1^; DL: 2 cfu mL^−1^; Response time: 2 min incubation with bacteria at 37 °C; Stability: 3 months.	*S. aureus, E. coli*, and *S. lentus* not interfering	Spiked water and apple juice samples	[96]
*Salmonella enterica serovar Typhimurium*	Ab/Porous graphene Detection by faradaic EIS with ferri/ferro	Range: 25–10^5^ cfu mL^–1^; DL: 13 cfu mL^–1^; Response time: 22 min; Stability: change of 3.36% in Rct after 7 days at −20 °C	*Bacillus cereus, E. coli O157:H7, Listeria monocytogenes, Pseudomonas aeruginosa,* and *Staphylococcus aureus* not interfering	Chicken broth	[132]
*Salmonella Typhimurium*	APT/rGO-CNT/GCE.	Range: 10^1^–10^8^ cfu mL^−1^; DL 10^1^ cfu mL^−1^; Test time: 10 min; Stability: 20 days in water at 4 °C	*E. coli, K. pneumonia, p. aeruginosa, S. aureus,* and *Enterococcus faecalis* not interfere, but *Salmonella enterica* such as *S. Albany*, *S. Enteritidis*, *S. Weltevreden*, *S. Typhi*, and *S. Derby* interfere	Raw chicken meat	[131]
	Detection by DPV using [Fe(CN)_6_]^4−3−^				
*E. coli O78:K80:H11*	APT-PLL-Bridged rebar graphene Detection by faradaic EIS using [Fe(CN)_6_]^4−/3−^ as a redox probe	Range: 10^1^–10^6^ cfu mL^−1^; Detection limit: 10^1^ cfu mL^−1^	*E. coli* DH5α, *p. vulgaris*, L. monocytogenes, *S. boydii*, *S. flexneri*, *E. aerogenes*, *C. braakii*, and *B. Subtilis* not interfering	Spiked fruit juice (guava, litchi, mango) and milk	[135]
*E. Coli*	rGO-FET, passivated with an ultrathin layer of Al_2_O_3_, then decorated with AuNPs onto which a specific Ab was immobilized	Range: 10^3^ to 10^5^ cfu mL^−1^; No significant change after 14 days at 4 °C; Response time: 50 s	Salmonella typhimurium and Streptococcus pneumonia are not interfering	Spiked river water; DL: 10^4^ cfu mL^−1^	[136]
*Botulinum neurotoxin serotype A*	Ab/Au-Gr-Cs/GCE Detection by faradaic EIS using [Fe(CN)_6_]^4–/3–^	Range: 0.27–268 pg mL^−1^; DL: 0.11 pg mL^−1^; Incubation time with the toxin: 60 min at 37 °C. Stability: 90% of response after 4 days at 4 °C in 0.1 MPBS buffer pH 7.4 with 0.02% NaN_2_	BoNT serotypes E and B	Spiked milk and human serum, BoNT was not detected in an unspiked sample by ELISA	[133]
*Botulinum neurotoxin A*	SNAP−25- peptide/chemically reduced GO/Au; Detection of BoNT serotype A light chain protease activity by DPV using [Fe(CN)_6_]^4–/3^	Range: 8.6 pg mL^−1^–1 ng mL^−1^; DL: 8.6 pg mL^−1^	BoNT serotype B lightchain (BoNT-LcB) and heat-treated LCA (denatured enzyme) did not interfere.	Spiked skimmed milk	[116]
*Staphylococcal enterotoxin B (SEB)*	Ab1/GR–AuNPs/GCE + Fc–Ab2.. Detection by SWV of ferrocene	DL: 5 ng mL^−1^; Analysis time: 35 min; Stability: 3 weeks at 4 °C	BTX−2, Okadaic acid MC-LR; Na^+^, K^+^, Ca^2+^, Mg^2+^, Sr^2+^, Cl^−^, SO_4_^2−^, Br^−^, HCO_3_^−^, and F^−^ not interfering. −95% cross-reactivity for BTX−1 and BTX−3	Spiked mollusk extract of *S. constricta, M. senhousia, and T. granosa,*	[128]
*Staphylococcal enterotoxin B*	APT/cDNA/rGO- AuNUs.; Detection of hematoxylin intercalator by DPV	Range: 5.0–500.0 fM; DL: 0.21 fM	Staphylococcal enterotoxin A, Staphylococcal enterotoxin C, BSA, and tryptophan not interfering	Spiked and simulated samples: milk, meat, and human serum	[129]
*Botulinum Toxin E*	Ab1/Gr-GCE + Ab2 + IgG-ALP/AuNPs. Detection by LSV–stripping of Ag deposited in the reaction of 3-IP and Ag^+^ catalyzed by ALP	Range: 10 pg mL^−1^–10 ng mL^−1^; DL: 5.0 pg mL^−1^; Analysis time: 65 min	BoNT/A, BoNT/B, and BoNT/F not interfering	Spiked orange juice and milk	[130]

TCID50: Median Tissue Culture Infectious Dose; AuNU: Au nano-urchins. ALP: alkaline phosphatase. LSV: Linear Sweep Voltammetry. 3-IP: 3-indoxyl phosphate. BoNT/A, BoNT/B: Botulinum toxin A and B. Fc-Ab2: ferrocene-labeled antibodies. BTX2: brevetoxin 2. SNAP25: synaptosomal-associated protein 25. Gr-graphene. APT: aptamer. Ab: antibody. cDNA: capture DNA.

**Table 2:** Examples of 2D-nanomaterial-based biosensors for the detection of mycotoxins.

Analyte	Description/Immobilization Details	Anal Performance	Selectivity	Application	Ref
Zearalenone	APT/AuNP/*p*-PtNTs/Au + Thionine-labeled GO Detection of thionine by DPV	Range: 5 × 10^−13^–5 × 10^−7^ g mL^−1^; DL: 1.67 × 10^−13^ g mL^−1^; Stability: decrease of 5.3% after storage for 7 days at 4 °C.	Aflatoxin B1, deoxynivalenol and patulin not interfering	Spiked maize extract	[97]
Fumonisin B1	cDNA/AuNP/GCE + APT/Gr-Th. Detection of thionine by cyclic voltammetry	Range: 1–10^6^ pg mL^−1^; DL: 1 pg mL^−1^; Incubation time with FB1: 25 min; Stability: no change in activity after 21 days at 4 °C	Fumonisin B1, Ochratoxin A, zearalenone and not interfering	Feed sample spiked at 5 concentration levels	[102]
Aflatoxin B1 (AFB1)	AFB1-BSA/AuNP/GCE + Ab/PtNP-CoTPP-rGO; Detection of H_2_O_2_ reduction by DPV	DL: 5.0 pg mL^−1^; Incubation time with sample and Ab-nanostructure conjugates: 25 min	Aflatoxins G1, FG2, alpha-fetoprotein, and thyroid-stimulating hormone not interfering. AFB2 interferes due to the cross-reactivity of the AFB1 antibody.	Spiked peanut and naturally contaminated peanut	[113]
Zearalenone	Ab1- *N*-GS/GCE + NP-PtCo-Ab2. Detection of H_2_O_2_ by amperometry at −0.4 V in phosphate buffer pH 5.8	Range: 0.005–25 ng mL^−1^; DL: 2.1 pg mL^−1^; Incubation time: NA; Incubation time with NP-PtCo-Ab2: 1 h; Stability: decrease of 5.6% of activity after after7-days in phosphate buffer pH = 5.8 at 4 °C.	Aflatoxin, ochratoxin, zeranol, kanamycin, gentamicin not interfering	Spiked pig feed	[137]
Aflatoxin B1	APT/c-PS/PDDA/Gr/GCE Detection by EIS	Range: 0.001–0.1 ng mL^−1^; DL: 0.002 ng mL^−1^; Stability: decrease to 85% response after 30 days at 4 °C	Ochratoxin A not interfering	Spiked oil and soy sauce	[112]
Aflatoxin B1	Ab/PPy-PPa-rGO Direct detection by non-faradaic EIS	Range: 10 fg mL^−1^–10 pg mL^−1^; DL: 10 fg mL^−1^; Incubation with AFB1: 50 min	Fumonisin B2, Aflatoxins G1 and G2, deoxynivalenol, and ochratoxin A A not interfering	Spiked corn	[138]
Aflatoxin B1	Ab/rGO–Ni. Detection by DPV using [Fe(CN)_6_]^3−/4−^	Range: 1–8 ng mL^−1^; DL: 0.16 ng mL^−1^; Stability: less than 10% decrease in signal after 6 weeks at 4 °C.	OTA not interfering	N/A	[89]
Ochratoxin A	APT/DNA1/Au + DNA2/AuNP–rGO Detection by EIS, using [Fe(CN)_6_]^4−/3^^−^	Range: 1 pg mL^−1^–50 ng mL^−1^; DL: 0.3 pg mL^−1^ (0.74 pM); Incubation time: 120 min (with-OTA). +60 min (with AuNPs–rGO-DNA2)	Ochratoxin B, Fumonisin B1 are not interfering	Spiked wines	[103]
Ochratoxin A	OTA-BSA/GCE + Ab-GO-PAMAM-Mn^2+^ Oxidation of 4-chloro−1-naphthol to an insoluble product. Detection by EIS using [Fe(CN)_6_]^4−/3^^−^	Range: 0.1 pg mL^−1^–30 ng mL^−1^; DL: 0.055 pg mL^−1^; Incubation time: 25 min with OTA and *anti*-OTA-GO-PAMAM-Mn^2+^, 1 min with KMnO_4_ and 25 min with 4-chloro−1-naphthol; Stability: 93.5% of activity after 20 days at 4 °C.	Ochratoxin B, Aflatoxin B1, B2 and G1; Na^+^, Cu^2+^, Fe^3+^, Mn^2+^, Zn^2+^, K^+^, Ca^2+^, Mg^2+^ not interfering	Spiked red wine	[139]
Aflatoxin B1	APT/GCE + rGO Detection by DPV using [Fe(CN)_6_]^4−/3−^	Range: 0.5 nM−4 μM; DL: 0.07 nM; Incubation time: 1 h with AFN1 +1 h with rGO; Stability: decrease of less than 4% in response after 1 week at 4 °C.	NA	Spiked pasteurized cow milk and human blood plasma spiked	[91]
AFB1	MB-APT/COOH-GO/SPCE. Detection of MB by DPV	Range: 0.05–6.0 ng mL^−1^; DL: 0.05 ng mL^−1^; Incubation time with AFB1: 1 h	AFM1, OTA not interfering	Spiked beer and wine	[111]
OTA	APT/Au-ATP-rGO/Au Detection by EIS using [Fe(CN)_6_]^4−/3−^	Range: 0.1–200 ng mL^−1^; DL: 0.03 ng mL^−1^; Incubation time with OTA: 90 min.; Stability: 1 month at 4 °C	Fumonisin B1, ochratoxin B not interfering	Spiked wine	[98]
OTA	APT/COOH-GO/SPCE. +Nanoceria (nCe)-OTA; Detection of H_2_O_2_	Range: 0.15–50 nM; DL: 1 nM	OTB not interfering	Cereal	[110]
OTA	APT/STR/GR/ITO. Detection by DPV using [Fe(CN)_6_]^4−/3^^−^	Range: 0.01–1000 ng mL^−1^; DL: 1 fg mL^−1^ (buffer); 10 pg mL^−1^ (spiked sample); Incubation with OTA: 8 min; Stability: 91.4% of initial activity after 7days	Malathion and heavy metals tested; malathion appears to interfere	Spiked grape juice	[105]
OTA	APT/cDNA/Au + g-C3N4. Detection −0.8 V in the presence of H_2_O_2_, based on the peroxidase-like activity of g-C3N4.	Range: 0.2–500 nM; DL: 0.073 nM; Incubation with OTA: 1 h Incubation with 1 mg/mL g-C_3_N_4_ solution: 30 min	Ochratoxin B and aflatoxin B1 not interfering	Spiked red wines, juice and corn	[140]
OTA	Ab/AuPdAg/MoS_2_/rGO/GCE.; Detection by DPV using [Fe(CN)_6_]^3−/4^^−^	Range: 10 fg mL^−1^–150 ng mL^−1^; DL: 5 fg mL^−1^; Incubation with OTA: 40 min; Stability: 99.6% of activity after 10 weeks; storage conditions not specified	Ochratoxin B, aflatoxin B1 IgG and glucose not interfering	Spiked coffee and corn	[141]
OTA	APT/cDNA/Au + MoS_2_ as peroxidase mimic Detection by amperometry at −0.2 V	Range: 0.5 pg mL^−1^–1.0 ng mL^−1.^; DL: 0.23 pg mL^−1^; Incubation time: 25 min at 37 °C with OTA + 25 min at RT with MoS_2_ + 5 min with H_2_O_2_ and hydroquinone; Stability: 90% of activity after 21 days at 4 °C.	Ochratoxins B and C, Aflatoxins B1 and B2, Cu^2+^, Mg^2+^, Zn^2+,^ and Mn^2+^ not interfering	Spiked red wine	[104]
OTA	MB-APT/β-CD/Au + MoS_2_/AuNP + ferrocenecarboxylic acid; Detection by DPV of MB and Fc	Range: 0.1 nM and 50 nM; DL: 0.06 nM; Incubation time with the aptamer: 1 h; Stability: 95.4% after 30 days of storage in the dark at 4 °C.	Aflatoxins M1 and B1, fumonisin M1, Ochratoxin B and C not interfering	Spiked red wine	[92]
Ochratoxin A	APT/AuNPs/MoSe_2_/GCE + cDNA + MB; Detection of MB by DPV	Range: 0.0001–1 nM; DL: 0.8 pM; Test time: 45 min; Stability: 3.2% decrease in activity after 15 days at 4 °C	Ochratoxin B and C and aflatoxin B1 not interfering	Spiked red wine	[142]
Aflatoxin B1	3DOM MoS2/AuNPs/Au aptamer-including tetrahedral DNA nanostructures + HRP-cDNA/AuNP-SiO_2_@Fe_3_O_4_ + thionine. Detection by DPV	Range: 0.1 fg mL^−1^–0.1 μg mL^−1^; DL: 0.01 fg mL^−1^; Incubation time with AFB1: 50 min at 37 °C; Incubation with HRP-cDNA/AuNP-SiO_2_@Fe_3_O_4_: 2.5 h Stability: 91% of initial activity after 1 month at 4 °C.	Aflatoxin B, M1, zearalenone and ochratoxin A are not interfering.	Spiked rice and wheat powder	[99]
OTA	b-APT/cDNA/AuNP-MoS_2_/GCE + STR- AuNP@Cd-MOF−74; Detection of Cd^2+^ by DPV	Range: 0.05–100 ng mL^−1^; DL: 10 pg mL^−1^; Incubation time with OTA: 25 min	Microcystin (MC)-LR MC-RR), thrombin, and Ochratoxin B not interfering	Spiked red wines	[143]

**Table 3:** Examples of biosensors including 2D nanomaterials for the detection of marine toxins.

Analyte	Description/Immobilization Details	Analytical Performances	Selectivity	Application	Ref
Mycrocystin LR (MC-LR)	APT/Commercial graphene-modified screen-printed electrode; Detection by SWV using [Fe(CN)_6_]^4–/3^^−^	Range: 1.9 pM–1.0 nM; DL: 1.9 pM; Stability: 2.9% decrease in activity after 1 month at 4 °C; Incubation time with MC-LR: 45 min	Okadaic acid, microcystin-LA, and microcystin-YR not interfering	Spiked fish extract and spiked tap water	[107]
MC-LR	APT/BiOBr nanoflakes/*n*-doped graphene/ITO; Photoelectrochimical biosensor	Range: 1 pM–100 nM; DL: 3.3 × 10^2^ pM; Incubation time with MC-LR: 30 min; Stability: no change in activity after 2 weeks at 4 °C	MC-LA, MC-YR not interfering	Spiked fish extract	[108]
Cylindrospermopsin	APT/Thionine–graphene; Detection by EIS using [Fe(CN)_6_] ^4–/3−^	Range: 0.39−78 ng mL^−1^ (1–200 nM); DL: 0.117 ng mL^−1^ (300 pM); Incubation time with CYN: 2 h; Stability: 88.2% and 74.7% of activity after 14 days and 30 days, respectively of storage in buffer at 4 °C	MC-LR, okadaic acid not interfering	Spiked lake water	[109]
Brevetoxin i	Ab-magnetic beads + BTX2-BSA-GGNR+ MCPE; Detection by SWV	Range: 1.0 pg mL^−1^–10 ng mL^−1^; DL: 1.0 pg mL^−1^; Incubation time with BTX2: 30 min	-Okadaic acid, MC-LR, Na^+^, K^+^, Ca^2+^, Mg^2+^, Sr^2+^, Cl^−^, SO_4_ ^2−^, Br^−^, HCO_3_^−^, and F^−^ not interfering. -BTX1, BTX3 interfere due to the cross-reactivity of the antibody for BTX2	Spiked mollusks extracts	[144]
Saxitoxin	Ab/Graphene nanosheets—lipid films; Detection by potentiometry.	Range: 1 × 10^−9^ M∓1 × 10^−6^ M; DL: 1 nM; Response time: less than 20 min	Mg^2+^, Ca^2+^, HC^3−^, SO_4_^2−^, Cl^−^, NO^3−^, NH_4_^+^ not interfering	Lake water and shellfish samples; Spiked mussels, oysters, and mollusks	[145]
Okadaic Acid	Ab/GSPE + okadaic acid-ovalbumin conjugate; Detection by SWV using [Fe(CN)_6_] ^4−/3^^−^	Range: up to 5000 ng L^−1^; DL: 19 ng L^−1^; Stability: 98% of activity after 40 days at 4 °C	Microcystin-LA not interfering	Spiked mussel extract	[146]
MC-LR	Ab-GO-IL -Au NP/GCE; Detection by DPV using [Fe(CN)_6_]^4−/3^^−^	Range: 0.1–1000 ng mL^−1^; DL: 0.1 ng mL^−1^; Incubation time with MC-LR: 50 min; Stability: 90.58% after 30 days in buffer at 4 °C	MC-LA, MC-RR, and MC-YR, as Na^+^, Ca ^2+^, K^+^,Cl^−^, and CO_3_ ^2−^ not interfering	Spiked river water	[147]
MC-LR	APT/AgI-NG/ITO; Photoelectrochemical aptasensor,	Range: 0.05 pM–5 nM; DL: 0.017 pM; Incubation time with MC-LR: 20 min; Stability: 94.5% of activity after 2 weeks at 4 °C	MC-LA and MC-YR are not interfering	Spiked fish	[148]
MC-LR	Ab/oxidized CVD graphene; Detection by EIS	Range: 0.005–10 μg L^−1^; DL: 2.3 ng L^−1^; Stability: 92.5% and 83.6% of activity after 1 and 2 weeks of storage at 4 °C.	Environmental water samples with TOC 0.53–8.99 mgL^−1^, total dissolved solid 118–170 mgL^−1^, sodium, (2.62–7.46 mgL^−1^), magnesium (4.9–14.67 mgL^−1^), aluminium (0.25–0.47 mgL^−1^), potassium (0.02–3.56 mgL^−1^), calcium (2.29–2.93 mgL^−1^), manganese (0.49–14.32 µgL^−1^), iron (0.86–174.2 µgL^−1^), copper (1.19–7.99 µgL^−1^) are not interfering	Spiked tap water, pond water, and lake water	[114]
MC-LR	APT/GO-modified printed electrode; (aptamer adsorbed or covalently immobilized; Detection by SWV using [Fe(CN)_6_]^4−/3^^−^.	Apt/Phys−300 μm; Range: 0.1 nM–1.0 μM DL: 0.038 nM; Apt/Phys−0.22 μm; Range: 0.1 nM–1.0 μM; DL: 0.088 nM; Apt/Cov−300 μm; Range: 1 nM−1.0 μM; DL:0.25 nM; Apt/Cov−0.22 μm: Range: 1 nM−1.0 μM; DL: 0.018 nM	Okadaic acid, MC-LA not interfering	N/A	[149]
Microcystin-L vccccR	MC-LR/AuNP@MoS2-TiONB/GCE + biotin-cDNA + Avidin-HRP; Detection by DPV	Range: 0.005–30 nM; DL: 0.002 nM; Incubation time: 120 min (60 min with the mixture of cDNA and MC-LR and 60 min with avidin -HRP; Stability: 90% activity after 10 days at 4 °C.	MC-LA, MC-YR, atrazine, and trichlorfon are not interfering	Spiked tap water, reservoir water and river water	[21]
MC-LR	Ab1/AuNR/MoS2/+HRP-Ab2; Detection by DPV	Range: 0.01–20 μg L^−1^; DL: 5 ng L^−1^; Incubation time with MC-LR: 1 h at 37 °C. and incubation with HRP-anti-MC-LR: 1 h at 37 °C; Stability: 99.46% and 95.62% after 1 week and 4 weeks of storage at 4 °C, respectively.	MC-RR, Okadaic acid, starch, ascorbic acid, Na^+^, NH^4+^, Ca^2+^, Cl^−^, SO_4_^2−^, and CO_3_^2−^ not interfering	Spiked: lake water, tap water, and drinking water	[150]
MC-LR	Ab/BSA-stabilized Au nanoclusters/MoS2/Au electrode: +Au@Pt core-shell nanoparticles Detection by DPV	Range: 1.0 ngL^−1^–1.0 mgL^−1^; DL: 0.3 ngL^−1^; Incubation time with MC-LR: 1 h at 37 °C. and incubation with Au@Pt: 1 h at 37 °C; Stability: 98% and 92% of initial activity after 1 week and 4 weeks storage at 4 °C	MC-RR, MC-LA, dopamine, uric acid, ascorbic acid, Al^3+^, Ca^2+^, Mg^2+^, Na^+^, K^+^, NH^4+^, SO_4_^2−^, CO_3_^2−^, NO^3−^ not interfering	Spiked water samples Recoveries: 99.6–101.3%	[151]
Okadaic acid	APT/phosphorene-gold NP/SPCE Detection by DPV using [Fe(CN)_6_]^3−/4^^−^	Range: 10 nM−250 nM; DL: 8 pM		Spiked mussel extract	[152]

**Table 4:** Examples of biosensors including 2D nanomaterials for the detection of other contaminants in food and the environment.

Analyte	Description/Immobilization Details	Anal Perform	Selectivity	Application	Ref
Gliadin	Ab/prGO/GCE Detection by DPV using [Fe(CN)_6_]^3−/4^^−^	Range: 1.2–34 ng mL^–1^; DL: 1.2 ng mL^−1^; Stability: 5% decrease in activity after 2 months at 4 °C	Lysozyme, casein, rice flour, cornflour not interfering	Wheat flour, pasta, cereal, Quaker oats, Gluten-free wheat flour Spiked rice flour and gluten-free flour	[153]
Ovalbumin	Ab/Graphene-modified SPCE Detection by DPV using [Fe(CN)_6_]^4−/3^^−^	Range: 1 pg mL^−1^ - 0.5 μg mL^−1^; DL: 0.83 pg mL^−1^; Incubation time with ovalbumin: 45 min; Stability: less than 2% decrease in activity after 14 days at 4 °C.	β-lactoglobulin, BSA, egg lysozyme, and casein are not interfering	Spiked cake extract %	[115]
β-lactoglobulin	Ab adsorbed/GO-modified electrode and Ab covalent/GO-modified electrode. Detection by SWV using [Fe(CN)_6_]^4−/3^^−^	Ab/Phys−300 μm; Range: 0.001–1.0 μg mL^−1^; DL: 0.46 ng mL^−1^; Ab/Phys−0.22 μmL; Range: 0.001–1.0 μg mL^−1^; DL: 0.79 ng mL^−1^; Ab/Cov−300 μm: Range: 0.01–1.0 μg mL^−1^; DL: 2.6 ng mL^−1^; Ab/Cov−0.22 μm: Range: 0.01–1.0 μg mL^−1^; DL: 1.2 ng mL^−1^	OVA, BSA not interfering	N/a	[149]
Catechol, Phenol, BPA	Tyrosinase/Silk peptide–graphene nanosheets/GCE Detection by amperometry at −0.10 V in PBS buffer pH 6 and 35 °C	Catechol; Range: 0.001–16.91 μM; DL: 0.23 nM-Phenol: Range: 0.0015–21.12 μM; DL: 0.35 nM–BPA: Range: 0.002–5.48 μM DL: 0.72 nM; Stability: 93.6% of activity after one month at 4 °C	Vitamin C, uric acid, m-dihydroxybenzene and *p*-nitrophenol not interfering. The sensor responds to dopamine, catechol, and phenol, besides BPA	BPA in plastic drinking bottles	[154]
BPA	Tyrosinase–graphydyne–chitosan/GCE Detection by amperometry at −0.04 V in 0.05 M PBS pH 7.0	Range: 0.0 × 10^−7^ to 3.5 × 10^−6^ M; DL: 24 nM; Response time: 20s; Stability: 94% activity after 3 weeks when stored dry at 4 °C	Phtalates (dimethyl, octyl) and Bisphenol S (BPS) are not detected	Water bottle (PC);beverage bottle (Al); coffee spoon (PP);beverage bottle (tinplate), mineralvwater bottle (PET); tap water	[35]
BPA	Tyrosinase–hydrophilic nanographene–chitosan/GCE. Detection by amperometry at −0.1 V	Range: 0.1–2 µM, DL: 33 nM	Phthalates, dimethylphthalate, doctylphthalate KNO_3_, sodiumcitrate, sodium oxalate, urea, ethylacetate, diethylcarbonate, acetonitrile, *n*-hexane, benzene, hexachlorobenzene, naphthalene are not interfering	5 samples: polycarbonate (PC) bottle, paper cup, PEGT water bottle and glass bottle.	[118]
BPA	Tyrosinase–graphene–Au/GCE. Detection by DPV, 0.1 M PBS pH 7	Range: 0.025–3 µM, DL: 1 nM	NA	Spiked plastic cup and milk carton samples	[155]
BPA	APT/Au NP-G/GCE Detection by CV using ferricyanide	Range: 0.01 and 10 µM; LOD: 5 nM; Incubation time: 30 min; Stability: 2 weeks at 4 °C	BPB, 4,4′-biphenol and 6F-BPA are not interfering	Spiked milk	[100]
BPA	Tyrosinase-(rGO–DAPPT/GCE Detection by amperometry at 0.1 V in 0.1 M phosphate buffer (pH 7.0)	Range: 1.0 × 10^−9^–3.8 × 10^−5^ M DL: 3.5 × 10^−10^ M; Stability: 90% of initial activity after 1 month at 4 °C	m-dihydroxy-benzene, *p*-nitrophenol, ascorbic acid, and uric acid not interfering but, *p*-dihydroxybenzene, dopamine, phenol, catechol, and cysteine interfere at 50 times larger concentrations than BPA	Spiked plastic drinking bottles	[156]
BPA	LACC/rGO-Fe_3_O_4_; Detection by amperometry at +0.15 V	Range: 6–228 ppb; DL:18 nM(4 ppb); Storage: 18% decrease in current density after 1 month in buffer at 4 °C	Catechol, ascorbic acid uric acid, 1-naphthol 4-nitrophenol and benzene not interfering; Glucose interferes	Spiked bottled water	[157]
Bisphenols	Tyrosinase-chitosan-CuMOF/GCE:. Detection by amperometry at −0.1 V in 0.05 M PBS buffer pH 7	BPE: Range: 5.0 × 10^−8^–3.0 × 10^−6^ M.; DL: 15 nM–BPF: Range: 5.0 × 10^−8^–3.0 × 10^−6^ M, DL:16 nM–BPA: Range: 5.0 × 10^−8^–3.0 × 10^−6^ M; DL: 13 nmM; BFB; Range: 1.25 × 10^−7^–8.0 × 10^−6^ M; DL: 56 nM–BFZ: Range: 2.5 × 10^−7^–5.0 × 10^−6^ M, DL: 33 nM	81.8–98.7% activity when the biosensor was incubated 0.5 with 0.1 mM of Hg2^+^, Pd2^+^, Cu2^+^, Fe2^+^, Co2^+^, Ba2^+^, Zn2^+^, Cd2^+^, and Ni2^+^ due to enzyme inhibition	metals	[158]
BPA	3D-CuMOF tyrosinase. Detection by amperometry at −0.1 V	BPA: Range: 5.0 × 10^−8^–3.0 × 10^−6^ M; DL: 13 nM; Storage stability: 90% of activity after 3 weeks at 4 °C in PBS buffer	K^+^, Na^+^, NO^3−^, HPO_4_^2−^, Cl, and acetone, acetonitrile, methanol, ethanol not interfering- phenols chemicals (e.g., phenol and catechol) not used in PC products, and phthalates are not substrates of tyrosinase	Spiked plastics: water bottle (PC), nursing bottle (PP), coffee spoon(PP), mineral water bottle (PET)	[159]
Phenol	Tyrosinase–chitosn−2D NiZn/GCE; Detection by amperometry at −0.05 V	Range: 0.08–58.2 μM; DL: 6.5 nM; Storage stability: 93% of the original response after 5-weeks at 4 °C	K^+^, Mg^2+^, Ca^2+^, Fe^3+^, Zn^2+^, SO^42−^, PO^43−^, CO^32−^, NO^3−^, uric acid, ascorbic acid, glucose not interfering	Spiked tap water	[160]
17β-estradiol	APT/WS2 Au NPs/GCE; Detection by DPV using [Fe(CN)_6_]^4−/3^^−^	Range: 1.0 × 10^−11^–5.0 × 10^−9^ M; DL: 2.0 × 10^−12^ M; Incubation time with 17β-estradiol: 3 h	Naphthalene and 1-aminoanthraquinone are not interfering	Spiked river water and serum	[101]
Phenol	Tyrosinase/2D layered pnictogens (phosphorene, arsenene, antimonene, and bismuthene; Detection by amperometry at −0.005 V in 0.1 M PBS buffer pH 6.5	Antimonene- based biosensor: Range: 500–2500 nM and 7.5–27.5 μM; DL: 255 nM.	Ca, Mg, Cu, aniline, benzyl alcohol not interfering	Spiked tap water	[161]
Total polyphenol	LACC/GrQD/MoS_2/_SPCE; Detection by amperometry at +0.05 V in 0.1 M acetate buffer pH 5.00	Caffeic acid; Range: 0.38–100 µM; DL: 0.32 µM–Chlorogenic acid; Range: 0.38–8.26 μM; DL: 0.19 μM (-) Epicatechin; Range: 2.86–100.00 μM; DL: 2.04 μM; Stability: 85% of initial activity after 4 weeks at 4 °C		Wines	[125]
Histamine	Ab/Gr; Detection by EIS with [Fe(CN)_6_]^4−/3^^−^	Range: 6.25–200 ppm (56.25 μM–1.8 mM; DL: 3.41 ppm (30.7 μM); Incubation time: 30 min	Bovine serum albumine(BSA) goat serum, whey protein not interfering	Tuna broth samples	[162]
Hypoxanthine	XOD/Gr-TiO2./GCE Detecction by amperometry at 0.8 V	Range: 20−512 μM; DL: 9.5 μM; Stability: 77% of initial activity after 10 days at 4C in 0.05 M PBS, 50% activity after 30 days	-Uric acid, ascorbic acid, and glucose do not interfere—xanthine interferes	Pork meat is stored at room temperature for seven days	[126]
Monosodium glutamate	Ab/AuNP-MoS2-chitosan/GCE. Detection by DPV using [Fe(CN)_6_]^4−/3^^−^	Range: 0.05–200 μM; DL: 0.03 µM; Stability: 98.7% response after 15 days at 4 °C	Cysteine, arginine, aspartic acid, butylated hydroxyl toluene and bisphenol-A are not interfering	Spiked vegetable soup %	[163]
Glucose	Gox/Au- Ti3C2Tx MXene/GCE; Detection by amperometry at −0.402 V	Gox/Au- MXene/GCE: Range: 0.1–18 mM; DL: 5.9 µM; Gox/MXene//GCE; LR: 0.5–6 mM; DL: 100 μM; Storage: 93% activity after 2 months	Dopamine, uric acid, ascorbic acid not interfering	N/A	[119]
H_2_O_2_	HRP/Phosphorene/GCE; Detection by amperometry at −0.1 V in PBS buffer pH 7.2	Range: 5–275 µM; DL: 0.14 µM; K_m_^app^ = 164 µM; Stability:93% and 69% of activity after 7 and 15 days, respectively	Dopamine ascorbic acid and uric acid do not interfere	N/A	[123]
H_2_O_2_	HRP/MB/chitosan/MoS2/graphite fiber Detection by amperometry at −0.3 V in 0.1 M phosphate buffer pH 7.0	Range: 0.1 to 90 μM; DL: 30 nM; Stability: 89% of initial activity after 60 days at 4 °C in buffer	Ascorbic acid, uric acid, dopamine, Na^+^, K^+^, Mg^2+^, Ca^2+^, Cl^−^ are not interfering	N/A	[164]
H_2_O_2_	Hemoglobin/poly-l-lysine-black phosphorus/GCE; Detection by cyclic voltammetry	Range: 10–700 µM	Uric acid and ascorbic acid are not interfering	N/A	[121]
H_2_O_2_	HRP-MoS_2_–Gr/GCE Detection by amperometry at −0.08 V	Range: 0.2 μM–1.103 mM; DL: 0.049 μM; Stability: 91.5% and 84.2% of initial activity after 2 weeks and 1 month, respectively at 4 °C.	Ascorbic acid, dopamine, cysteine, and lysine do not interfere	N/a	[124]
H_2_O_2_	Cytochrome c/ZIF−8-MOFs/SPCE screen-printed electrode; Detection by amperometry at −0.05 V	Range: 0.09–3.6 mM	Glucose, dopamine, and bovine serum albumin are not interfering	Spiked milk and beer	[165]
Acetochlor	GOx/CS/NH2-MIL−125(Ti)/TiO2-MOF/GCE Photoelectrochemical sensor, inhibition of glucose oxidase	Range: 0.02–200 nM; DL: 0.003 nM. Stability: 92.5% activity after 30 days at 4 °C	Sucrose, glycine, citric acid, K^+^, Na^+^, Ca^2+^, prometryn, clethodim, cycloxydim, and sethoxydim not interfering	Spiked strawberry, tomato, cucumber, and greens	[122]
Fenitrothion	AChE-BSA/TMDs (MoS_2_, MoSe_2_, WS_2_, WSe_2_/GCE Detection by amperometry at 0.1 V	1T-Phase WS_2_ based biosensor; Range: 1–1000 nM; DL:2.86 nM; Incubation time: 5 min	Fe^2+^, Cu^2+^, ascorbic acid and phenol: not interfering	Spiked apple juice	[127]
Forchlorfenuron	Catalase/boron nitride/GCE; Detection by amperometry at −0.35 in 1 M phosphate buffer pH 7.0 V	Range: 0.5–10.0 µM; DL: 0.07 μM; Stability: 91.3% of initial activity after 2 months at 4 °C	Glucose, sucrose, glycine, citric acid, Na^+^ and Ca^2+^ not interfering	Spiked orange juice, kiwi, watermelon, grape	[120]
Methyl parathion	Nafion/AChE/MOF/electrode detection by DPV	[Fe-MOF-NH2]N2: Range 10^−12^–10^−8^ g mL^−1^; DL: 3.2 × 10^−13^ g mL^−1^ (1.2 × 10^−12^ M); Zr-MOF-NH_2_]N_2_: Range: 5.0 × 10^−13^–5.0 × 10^−9^ g mL^−1^, DL: 1.8 × 10^−13^ g mL^−1^ (6.9 × 10^−13^ M) [La-MOF-NH_2_]_N2_ Range: 1.0 × 10^−13^–5.0 × 10^−9^ g mL^−1^; DL: 5.8 × 10^−14^ g mL^−1^ (2.2 × 10^−13^ M) Incubation time: 12 min; Stability: 81%, 83% and 84% after 4 weeks at 4 °C in PBS buffer pH 7.	No data reported	N/a	[166]
Pb ^2+^	DNA functionalized iron-porphyrinic metal–organic framework ((Fe-*p*)*n*-MOF-Au-GR/Au-PWE. Detection by DPV	Range: 0.03–1000 nM.; DL: 0.02 nM; Stability: 95% and 50% of activity after 20 and 60 days at room temperature, respectively; 95% and 94% of activity after 60 days of storage in the refrigerator and freezer, respectively	Fe^3+^, Cd^2+^, Co^2+^, Zn^2+^, Mn^2+^, Ni^2+^, Cu^2+^, Hg^2+^ and Ag+ not interfering	Industrial waste water, river water, fruit juice (orange and apple), solid samples, serum	[167]

prGO: porous reduced graphene oxide. DAPPT: 1,3-di(4-amino-1-pyridinium) propane tetrafluoroborate ionic liquid. rGO-Fe_3_O_4_ NPs: hybrid conjugate of reduced graphene oxide/ferrous–ferric oxide nanoparticles. GrQD: graphene quantum dots. XOD: xanthine oxidase. AChE: acethylcholine esterase. Au-PWE: Au NP modified paper working electrode.
